# Solvent-induced dual nucleophiles and the α-effect in the S_N_2 *versus* E2 competition†

**DOI:** 10.1039/d4cp00671b

**Published:** 2024-03-15

**Authors:** Xiangyu Wu, F. Matthias Bickelhaupt, Jing Xie

**Affiliations:** a Key Laboratory of Cluster Science of Ministry of Education, Beijing Key Laboratory of Photoelectronic/Electrophotonic Conversion Materials, School of Chemistry and Chemical Engineering, Beijing Institute of Technology Beijing 100081 China jingxie@bit.edu.cn; b Department of Chemistry and Pharmaceutical Sciences, AIMMS, Vrije Universiteit Amsterdam De Boelelaan 1108 1081 HZ Amsterdam The Netherlands f.m.bickelhaupt@vu.nl; c Institute for Molecules and Materials (IMM), Radboud University Nijmegen Heyendaalseweg 135 6525 AJ Nijmegen The Netherlands; d Department of Chemical Sciences, University of Johannesburg Auckland Park Johannesburg 2006 South Africa

## Abstract

We have quantum chemically investigated how microsolvation affects the various E2 and S_N_2 pathways, their mutual competition, and the α-effect of the model reaction system HOO^−^(H_2_O)_*n*_ + CH_3_CH_2_Cl, at the CCSD(T) level. Interestingly, we identify the dual nature of the α-nucleophile HOO^−^ which, upon solvation, is in equilibrium with HO^−^. This solvent-induced dual appearance gives rise to a rich network of competing reaction channels. Among both nucleophiles, S_N_2 is always favored over E2, and this preference increases upon increasing microsolvation. Furthermore, we found a pronounced α-effect, not only for S_N_2 substitution but also for E2 elimination, *i.e.*, HOO^−^ is more reactive than HO^−^ in both cases. Our activation strain and quantitative molecular orbital analyses reveal the physical mechanisms behind the various computed trends. In particular, we demonstrate that two recently proposed criteria, required for solvent-free nucleophiles to display the α-effect, must also be satisfied by microsolvated HOO^−^(H_2_O)_*n*_ nucleophiles.

## Abstract 2

We have quantum chemically investigated how microsolvation affects the various E2 and S_N_2 pathways, their mutual competition, and the α-effect of the model reaction system HOO^−^(H_2_O)_*n*_ + CH_3_CH_2_Cl, at the CCSD(T) level.

## Graphical abstract

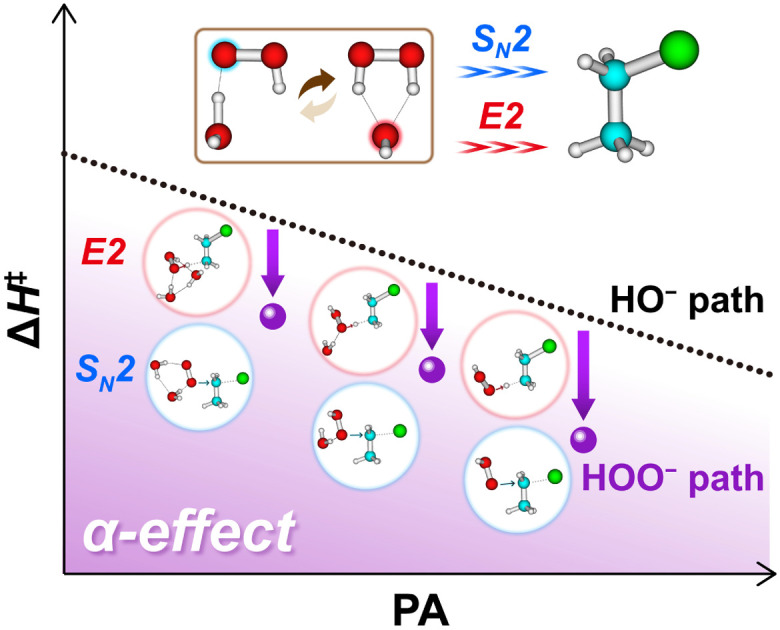

## Introduction

Bimolecular nucleophilic substitution (S_N_2) and elimination (E2) are ubiquitous, often mutually competing, reactions. For nearly 50 years, the mechanism of the S_N_2 reaction has been extensively studied experimentally and theoretically, including its temperature dependence,^1–3^ benchmark studies,^4–11^ steric effects,^12–21^ and solvent effects.^14,15,22–29^ Solvation can have a tremendous effect on chemical reactions.^14,15,22,30–32^ Rate constants, for example, can be reduced by up to 16 orders of magnitude from the gas phase to aqueous solution.^33^ Microsolvation bridges the gap between the gas phase and bulk solution and constitutes a powerful approach for obtaining a detailed understanding of how exactly solvent molecules affect reactions.^33–39^ Most research in this direction has been focused on microsolvated S_N_2 reactions of Y^−^(H_2_O)_*n*_ + CH_3_X.^1,23,26,30,32,34,40–50^ Significantly less attention has been paid to microsolvated E2 reactions^51–53^ or solvent effects on the S_N_2 *versus* E2 competition.^25,27,37,54–56^ Previous theoretical studies have shown that the stepwise introduction of solvent molecules (*e.g.*, HF,^25^ CH_3_OH^27^ and H_2_O^37,55^) as well as the increase in their solvation power favors S_N_2 relative to E2 pathways, and can lead to a switch from overall E2 to S_N_2 reactivity. The mechanism behind this solvation favoring the S_N_2 pathway is that S_N_2 reactions have a significantly lower characteristic distortivity and thus lower activation strain than E2 reactions and therefore suffer less from the reduced interaction between the nucleophile and the substrate when the nucleophile's basicity is attenuated upon solvation, as shown by Bickelhaupt, Hamlin *et al.*^55^

The peroxide anion HOO^−^ is an interesting nucleophile, because the monohydrated peroxide anion displays dual nucleophile character, where both HOO^−^(H_2_O) and (HOOH)(OH^−^) species are similarly stable.^57^ Moreover, as HOO^−^(H_2_O) reacts with CH_3_Cl,^58^ both HOO^−^ and HO^−^ anions are possible attacking nucleophiles, and this was observed in our recent direct dynamic simulation work.^36^ The introduction of water into the HOO^−^ nucleophile enriches the reaction dynamics for it adds the proton-transfer induced HO^−^-S_N_2 pathway; one can expect that if the substrate was ethyl halides, the E2 pathway will emerge and make the dynamics more complicated and interesting.

Besides, HOO^−^ is a typical α-nucleophile, possessing a lone pair of electrons adjacent to the nucleophilic atom. The term α-effect^59^ has been used to describe the enhanced reactivity of α-nucleophiles compared to that of normal nucleophiles by deviating from the Brønsted-type correlations found for normal nucleophiles.^60^ There has been extensive discussion on the origin of the α-effect, as well as a controversy about whether the α-effect is controlled by the intrinsic properties of the α-nucleophile or by external solvent effects.^61–63^ In terms of the intrinsic properties, mechanisms such as ground state destabilization, transition state stabilization, and thermodynamic product stability were proposed to be the origin of the α-effect.^63–69^ The α-effect has been observed in a variety of S_N_2 reactions,^58,61,70–74^ yet fewer studies have addressed its relevance to E2 reactions.^75,76^ A recent study by Hamlin *et al.*^77^ proposed two intrinsic criteria for the α-nucleophile to display the α-effect: (1) a higher energy HOMO and (2) a smaller HOMO lobe and overall amplitude of occupied orbitals on the nucleophilic center compared to the normal nucleophile. These criteria were proposed for solvent-free nucleophiles, and it is intriguing to examine whether they suit microsolvated nucleophiles.

**Scheme 1:**
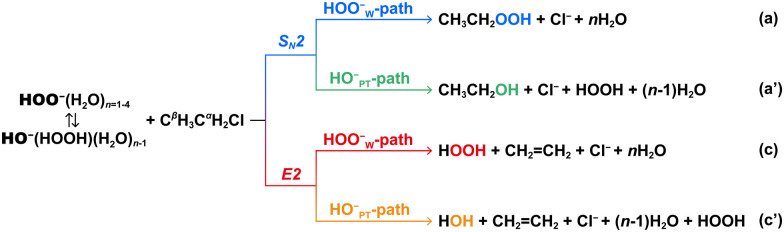
S_N_2 and E2 reaction pathways of HOO^−^(H_2_O)_*n*=1–4_ + CH_3_CH_2_Cl reactions. In pathway c′, one water is the conjugate acid of the nucleophile HO^−^.

In this study, we report a quantum chemical study on the HOO^−^(H_2_O)_*n*_ + CH_3_CH_2_Cl reaction (Scheme 1), where *n* = 0 to 4 is the number of water molecules. We explore the full reaction pathways that, after formation of the initial E2 or S_N_2 product complexes, lead to the separated products, as shown in Scheme 1. This is in agreement with earlier experimental and simulation studies on closely related microsolvated ion–molecule S_N_2 reactions, which have shown that the formation of the unsolvated ionic products strongly dominates the formation of the solvated ionic products because of dynamic bottlenecks which make solvent transfer from nucleophiles to leaving groups less likely.^45,47,78^ The purpose of this study is three-fold, namely, to investigate the effect of solvation on (1) the competing S_N_2 and E2 reaction pathways, (2) the competing normal HOO^−^-pathways and the solvent-induced HO^−^-pathways, and (3) the α-effect on both, the S_N_2 and E2 reactions, in terms of the nucleophiles' intrinsic properties.

## Results and discussion

### Potential energy surfaces

**Fig. 1:**
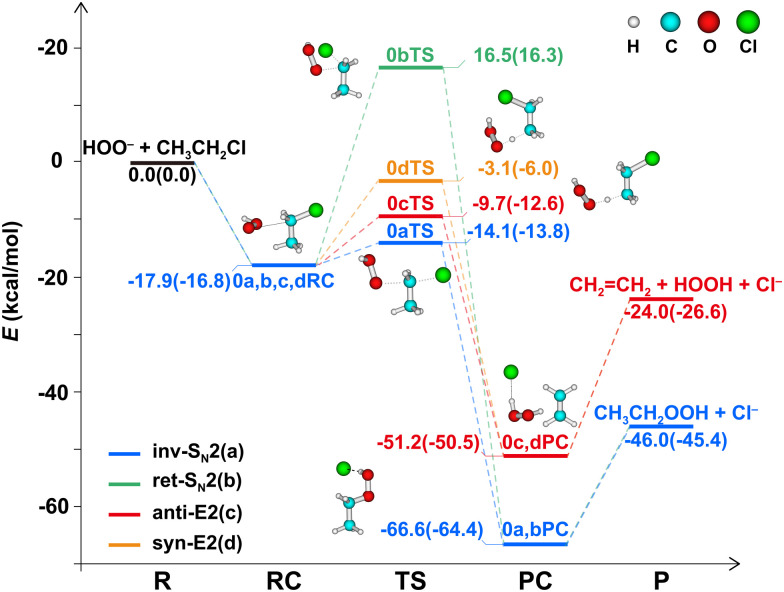
Potential energy profiles of the S_N_2 and E2 reactions of HOO^−^ + CH_3_CH_2_Cl using the CCSD(T)/aug-cc-pVTZ//MP2/6-311++G(d,p) method. Energies in the normal text and parentheses are the electronic energy and enthalpy at 298.15 K, respectively.

Fig. 1 depicts the potential energy surfaces (PESs) of the various S_N_2 and E2 mechanistic pathways of HOO^−^ reacting with CH_3_CH_2_Cl, all of which display the typical “double-well” shape in which reactants (R) combine to a reactant complex (RC) that is connected *via* a transition state (TS) to product complexes (PC) and eventually separate products (P). The S_N_2 reaction is significantly more exothermic than the E2 reaction, with respective reaction energies of −46.0 and −24.0 kcal mol^−1^. Depending on the site and spatial direction under which HOO^−^ attacks, the S_N_2 reaction proceeds either *via* backside substitution (inv-S_N_2, a) or *via* front side substitution that retains the stereostructure (ret-S_N_2, b),^79^ whereas the E2 reaction may take place either *via anti*-elimination (c) or *via syn*-elimination (d).^80^ For the inv-S_N_2 pathway, the nucleophile HOO^−^ attacks C^α^ from the back side of the leaving group and causes the CH_3_-moiety to undergo Walden inversion toward the product CH_3_CH_2_OOH. For the ret-S_N_2 pathway, HOO^−^ attacks C^α^ from the front side of the leaving group and the CH_3_-moiety retains its geometry in product CH_3_CH_2_OOH. For *anti*-E2 or *syn*-E2 pathways, HOO^−^ attacks a β proton from the opposite or same side of the leaving group, respectively, leading to the abstraction of this proton and the formation of the E2 products CH_2_ = CH_2_ + Cl^−^ + HOOH. The energy barriers decrease along ret-S_N_2 (16.5 kcal mol^−1^) > *syn*-E2 (−3.1) > *anti*-E2 (−9.7) > inv-S_N_2 (−14.1). The enthalpy and free energy values show the same trend (Table 1). The inv-S_N_2 and *anti*-E2 pathways have the lowest barriers and therefore outperform other reaction channels. Therefore, we now focus on these two reaction mechanisms and label them, for simplicity, as S_N_2 and E2.

**Table 1:** Calculated overall barriers (in kcal mol^−1^) for the S_N_2 and E2 paths of HOO^−^(H_2_O)_*n*_ + CH_3_CH_2_Cl reactions with the electronic energy (*E*), enthalpy (*H*, 298.15 K) and Gibbs free energy (*G*, 298.15 K) values reported^a^

	*n*	HOO^−^_W_-path			HO^−^_PT_-path		
		Δ*E*^‡^	Δ*H*^‡^	Δ*G*^‡^	Δ*E*^‡^	Δ*H*^‡^	Δ*G*^‡^
inv-S_N_2	0	−14.1	−13.8	−3.9	−14.0	−14.0	−5.4
	1	−4.6	−3.8	5.2	−1.4	−0.4	9.5
	2	0.6	1.7	13.2	2.7	3.8	16.3
	3	3.1	4.1	14.8	8.0	9.3	19.4
	4	8.0	8.3	18.2	10.0	11.0	22.4

*anti*-E2	0	−9.7	−12.6	−3.5	−12.7	−15.8	−8.0
	1	2.4	0.1	7.9	3.8	1.6	9.6
	2	7.9	6.9	19.2	8.8	6.9	17.4
	3	14.0	12.5	21.6	15.2	13.7	22.0
	4	17.9	16.4	25.1	18.2	16.2	26.9

ret-S_N_2	0	16.5	16.3	27.5	20.9	20.5	29.4
*syn*-E2	0	−3.1	−6.0	4.1	−5.2	−8.6	−0.5

a Computed at CCSD(T)/aug-cc-pVTZ//MP2/6-311++G(d,p).

It has been shown that hydrated peroxide anions, HOO^−^(H_2_O)_*n*_, tend to abstract a proton from H_2_O and form more stable species HO^−^(HOOH)(H_2_O)_*n*−1_ (Fig. S1, ESI†).^36,38,57,58^ Consequently, the solvent-induced proton-transfer HO^−^-moiety is a potential nucleophile to compete with the original nucleophile HOO^−^. Hence, when HOO^−^(H_2_O)_*n*_ reacts with CH_3_CH_2_Cl, four pathways are possible: (a) HOO^−^_W_-S_N_2, “W” indicates that the nucleophile is bound with water molecules; (a′) HO^−^_PT_-S_N_2, “PT” indicates that the HO^−^ nucleophile is induced by proton transfer from water molecules; (c) HOO^−^_W_-E2; and (c′) HO^−^_PT_-E2 (Scheme 1). Note that in the hydrated system, the HOO^−^_W_-E2 and HO^−^_PT_-E2 pathways generate the same products.

**Fig. 2:**
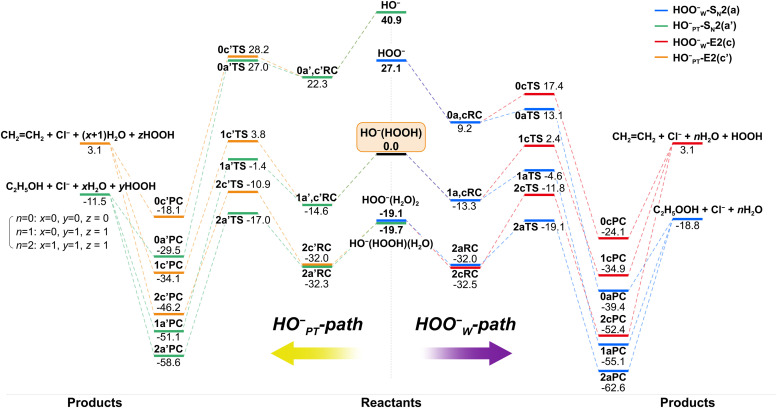
Potential energy profiles of HOO^−^(H_2_O)_*n*=0–2_ + CH_3_CH_2_Cl reactions for both, the HOO^−^_W_-path (right) and the HO^−^_PT_-path (left). Energies (kcal mol^−1^) are relative to HO^−^(HOOH) + CH_3_CH_2_Cl at the level of CCSD(T)/aug-cc-pVTZ//MP2/6-311++G(d,p) without the ZPE correction.

**Fig. 3:**
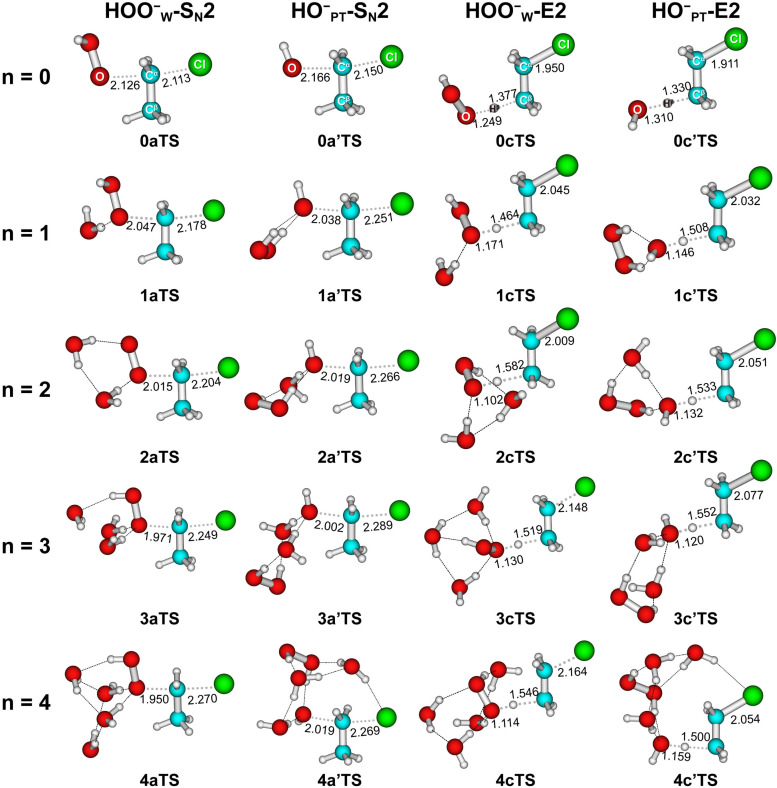
Structures of transition states of HOO^−^(H_2_O)_*n*=0–4_ + CH_3_CH_2_Cl optimized at MP2/6-311++G(d,p). Bond lengths are in angstrom.

To show the effect of individual solvent molecules, we plotted the potential energy profiles of the HOO^−^(H_2_O)_*n*=0,1,2_ + CH_3_CH_2_Cl reactions in Fig. 2 for both HOO^−^_W_-paths (right panel) and HO^−^_PT_-paths (left panel). We used 0, 1 and 2 as prefixes to denote the number of solvent molecules when naming the species. The corresponding transition state structures are shown in Fig. 3. The involvement of multiple H_2_O molecules complicates the structures, so the most stable structures of each species and corresponding energetics were used in the discussion. Information on higher-energy conformational isomers is provided in Fig. S2–S6 (ESI†) for interested readers. Using HO^−^(HOOH) + CH_3_CH_2_Cl as the reference point, as observed, the HOO^−^_W_-S_N_2 reactions (−18.8 kcal mol^−1^) are more exothermic than HO^−^_PT_-S_N_2 reactions (−11.5 kcal mol^−1^), where both are more exothermic than E2 reactions (3.1 kcal mol^−1^). The addition of water molecules to the ion–molecule system stabilizes each species. For the singly- and doubly-hydrated systems, the potential energy profiles of both S_N_2 and E2 reactions remain double-well shaped. However, due to the differential stabilization effect of water molecules on the reactants and transition states, the barrier heights changed differently for the S_N_2 and E2 reactions. The energetic values are presented in Table 1 and details will be discussed in the next section.

### Competition S_N_2 *versus* E2 and HOO^−^*versus* HO^−^

**Fig. 4:**
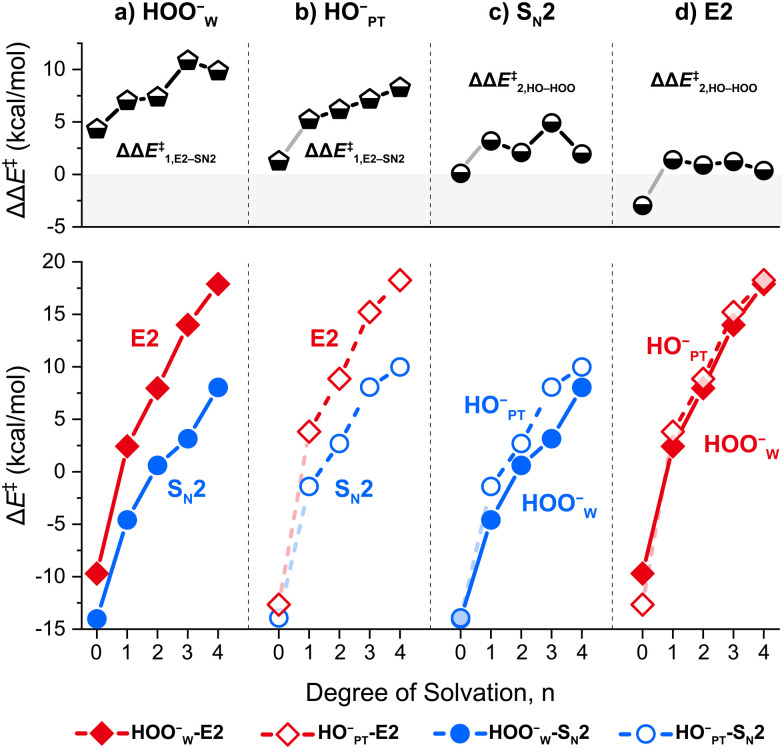
Barrier heights Δ*E*^‡^ (bottom) and differences in barrier heights ΔΔ*E*^‡^ between two paths (top) of the four mechanistic pathways of the model reaction system HOO^−^(H_2_O)_*n*=0–4_ + CH_3_CH_2_Cl, computed at CCSD(T)/aug-cc-pVTZ//MP2/6-311++G(d,p) without the ZPE correction. Note: for the HO^−^_PT_-path, *n* = 0 corresponds to the HO^−^ + CH_3_CH_2_Cl reaction.

The reaction barrier, Δ*E*^‡^ = *E*(TS) − *E*(R), of all four paths increases systematically as the extent of microsolvation (*i.e.*, the number of solvent molecules) increases, as shown in Fig. 4. This trend of an increasing barrier agrees well with the decreasing reaction rate observed previously in experimental studies of microsolvated ion–molecule reactions.^1,30,33,34,40,52,54,81,82^ Focusing on the competition between S_N_2 and E2 pathways (Fig. 4a and b), the barrier of E2 pathways increases slightly faster upon microsolvation than that of S_N_2 pathways. Thus, the extent to which the E2 barrier exceeds that of the S_N_2 barrier, *i.e.* ΔΔ*E*^‡^_1_ = Δ*E*^‡^(E2) − Δ*E*^‡^(S_N_2), increases upon microsolvation. For instance, as *n* increases from 0 to 4, the ΔΔ*E*^‡^_1_ value increases from 4.3 to 7.0, 7.4, 10.8, and 9.9 kcal mol^−1^ for the HOO^−^_W_-path. Consequently, the S_N_2 paths always dominate and they do so even more at higher degrees of solvation. This enlargement of the E2–S_N_2 barrier difference (ΔΔ*E*^‡^_1_) with a stepwise increase of microsolvation is also computed for the HO^−^_PT_-pathways, with corresponding values of 1.3, 5.2, 6.2, 7.2 and 8.3 kcal mol^−1^, respectively. Our finding consolidates earlier work that focused on the E2 and S_N_2 reactions of microsolvated model systems HO^−^(H_2_O)_*n*_ + CH_3_CH_2_X (X = Cl, Br, I) and F^−^ + CH_3_CH_2_F + *n*HF.^25,37,80^

Regarding the competition between the α-nucleophile HOO^−^_W_ and the normal nucleophile HO^−^_PT_ (Fig. 4c and d), the barrier height difference, defined as ΔΔ*E*^‡^_2_ = Δ*E*^‡^(HO^−^_PT_) − Δ*E*^‡^(HOO^−^_W_), is greater than zero for almost all cases, that is, the α-nucleophile reacts faster in almost all cases. The main trend is that introducing microsolvation, *i.e.*, going from *n* = 0 to *n* ≥ 1, enhances the α-effect, *i.e.*, ΔΔ*E*^‡^_2_. However, the dependence of the α-effect as a function of introducing more solvent molecules (*i.e.*, along *n* = 1, 2, 3, 4) is less uniform. For the S_N_2 paths, the barrier difference ΔΔ*E*^‡^_2_(S_N_2) ranges from 2.0 to 4.9 kcal mol^−1^, whereas there is a smaller difference between the E2 pathways (Fig. 4d), and ΔΔ*E*^‡^_2_(E2) ranges from 0.4 to 1.4 kcal mol^−1^, indicating that the HO^−^-E2 path can be strongly competitive to the HOO^−^-E2 pathway, provided sufficient energy is available to pass the E2 barriers.

In brief, among the four competing pathways, the HOO^−^_W_-S_N_2 path dominates with incremental solvation. In what follows, we seek the reason for barrier height difference upon solvation by analyzing the properties of nucleophiles and transition states.

#### Nucleophiles and HOMO–LUMO interactions

Besides the hydrated peroxide anion nucleophiles HOO^−^(H_2_O)_*n*=1–4_ and the associated PT-induced HO^−^(HOOH)(H_2_O)_*n*=1–3_ nucleophiles, we also considered the hydrated hydroxide anion nucleophiles HO^−^(H_2_O)_0–4_^37^ for comparison. The latter was labeled as the HO^−^_W_-path. The properties calculated include the energy level of the HOMO, the proton affinity (PA), and the ethyl cation affinity (ECA) of these nucleophiles.

**Fig. 5:**
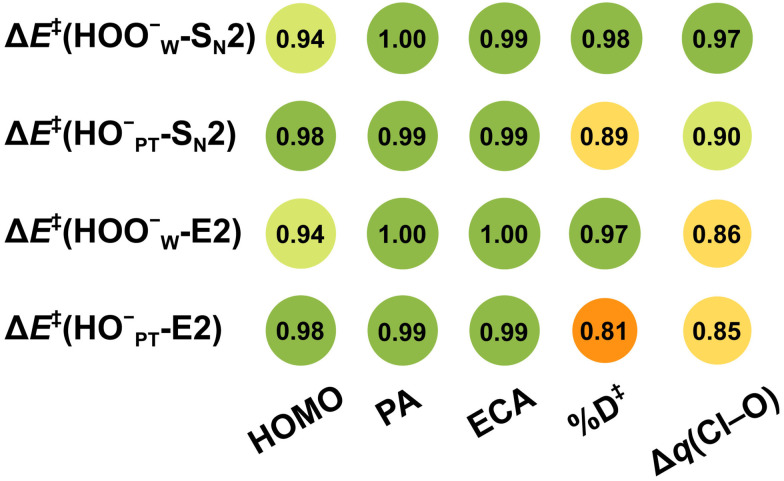
Correlation coefficients (*R*^2^) for the linear correlation between the E2 and S_N_2 barrier heights and the physical properties of the HOO^−^(H_2_O)_*n*=0–4_ + CH_3_CH_2_Cl reaction systems: nucleophile HOMO energy, PA and ECA, distortion parameter %*D*^‡^, and charge asymmetry Δ*q*(Cl–O).

In line with our previous studies,^25,38,55,80^ we found herein that microsolvation lowers the energy of the HOMO of both HOO^−^ and HO^−^ systematically upon adding an additional solvent molecule, either H_2_O or HOOH, by the HOMO–LUMO interaction with the σ_O–H_* (solvent) LUMO (Table S4, ESI†). The HOMO of the microsolvated peroxide anion in HOO^−^(H_2_O)_2,3,4_ (−5.6, −6.0, and −6.6 eV) always remains higher in energy than that of the equivalently microsolvated hydroxide anion in HO^−^(HOOH)(H_2_O)_1,2,3_ (−6.5, −6.9, and −7.3 eV) and HO^−^(H_2_O)_2,3,4_ clusters (−5.7, −6.6, and −7.2 eV; see Table S4, ESI†). As shown previously by Bickelhaupt *et al.*,^25,55^ this situation gives rise to a smaller HOMO–LUMO energy gap and a more stabilizing HOMO–LUMO interaction with the LUMO (substrate) (in this work, the substrate is CH_3_CH_2_Cl) and, therefore, to a lower barrier for the reactions of microsolvated HOO^−^ than for the corresponding reactions of microsolvated HO^−^. Indeed, we found a strong correlation of the barrier heights of the S_N_2 and E2 reactions with the HOMO energy level of the microsolvated peroxide and hydroxide nucleophiles (Fig. 5 and Fig. S8, ESI†). The same holds true for the related quantities of proton affinity (PA) and ethyl cation affinity (ECA), which are measures of the nucleophiles’ ability to bind with a proton and an ethyl cation. The PA and ECA values are defined as the enthalpy change of NuH → Nu^−^ + H^+^ and CH_3_CH_2_Nu → Nu^−^ + CH_3_CH_2_^+^, respectively (see Table S5 for all computed PA and ECA values, ESI†).

With the introduction of solvent molecules, the PA or ECA values of nucleophiles decrease continuously, that is, both types of affinities become weaker. Gratifyingly, the overall barrier heights (Δ*E*^‡^) of the S_N_2 and E2 reactions have, again, a strong linear correlation with the PA or ECA of the various microsolvated peroxide and hydroxide nucleophiles (*R*^2^ ≈ 1.00; see Fig. 5). In line with this, there is also a good to reasonable correlation between the height of the barrier and the amount of charge transferred from the nucleophile to the substrate, as computed with natural population analysis (NPA) atomic charges (*R*^2^ = 0.85–0.97; see Fig. 5 and Table S7, ESI†).^37,83^ Thus, a stronger charge transfer goes with a lower barrier.

#### S_N_2 *versus* E2 characteristic distortivity

In this section, we analyze how the barrier heights correlate with the geometrical characteristics of the transition states for the S_N_2 and E2 mechanistic pathways. As before, we focus on the pathway involving the geometrical configuration of solvent molecules that yields the most stable TS conformation for each reaction mechanism. In the first place, we recall that E2 elimination goes inherently with a higher characteristic distortivity than S_N_2 substitution which is a factor that contributes to a higher activation strain and thus a higher barrier. To put this on a more quantitative basis for the model reactions investigated herein, we have defined the distortion parameter %*D*^‡^, which depends linearly on the stretch of C^α^–Cl and H^β^–C^β^ bonds in the TS, as defined below:%*D*^‡^ = %C^α^Cl^‡^ + %H^β^C^β‡^
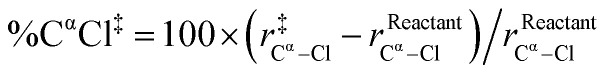

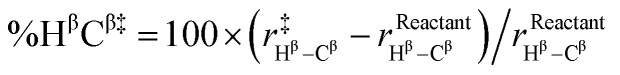


Indeed, we found a reasonably strong linear correlation of the S_N_2 and E2 reaction barriers with %*D*^‡^ (*R*^2^ = 0.81 to 0.98; see Fig. 5). The trend is that the more geometrically distorted the TS, the higher the barrier.

Furthermore, we found that incremental microsolvation makes the transition structures in general more product-like, as elaborated upon in the following. The S_N_2 pathway and the transition state involve the breaking of the C^α^–Cl bond and the formation of the O–C^α^ bond, whereas the E2 pathway and transition state involve the breaking of both the C^α^–Cl bond and the H^β^–C^β^ bond and the formation of the peroxide or hydroxide O–H^β^ bond (Fig. 3). Table 2 shows that, as the degree of microsolvation increases from 0 to 3, for transition structures of inv-S_N_2 reactions, the O–C^α^ bond shortens and the C^α^–Cl bond lengthens systematically. In the case of the transition structures of *anti*-E2 reactions, there are a few irregularities but, by and large, the O–H^β^ bond shortens, and both the C^α^–Cl bond and the H^β^–C^β^ bond lengthen upon going from the unsolvated to the microsolvated situation. In line with these structural characteristics, the Cl leaving group becomes increasingly negatively charged in the transition states of both S_N_2 and E2 reactions when the degree of microsolvation increases, as reflected by the computed NPA charges (see Table S7, ESI†). For example, the negative charge of the leaving group *q*(Cl) of the HOO^−^_W_ S_N_2 path increases from −0.539 to −0.675 a.u., and the value of the HOO^−^_W_ E2 path increases from −0.355 to −0.580 a.u. These findings are all consistent with the fact that the transition states become more product-like. Thus, as-stated above, microsolvation shifts the TS to a later, more product-like point along the reaction coordinate.

Selected bond lengths (in Å) of the transition structures

**Table:** 

HOO^−^_W_-path				HO^−^_PT_-path		
inv-S_N_2-TS				inv-S_N_2-TS		
*n*	*r*(O–C^α^)	*r*(C^α^–Cl)	*r*(H^β^–C^β^)	*r*(O–C^α^)	*r*(C^α^–Cl)	*r*(H^β^–C^β^)
0	2.126	2.113	1.094	2.166	2.150	1.091
1	2.047	2.178	1.094	2.038	2.251	1.091
2	2.015	2.204	1.094	2.019	2.266	1.092
3	1.971	2.249	1.094	2.002	2.289	1.092
4	1.950	2.270	1.094	2.019	2.269	1.092

**Table:** 

*anti*-E2-TS					*anti*-E2-TS			
*n*	*r*(O–H^β^)	*r*(C^α^–Cl)	*r*(H^β^–C^β^)	*r*(C^α^–C^β^)	*r*(O–H^β^)	*r*(C^α^–Cl)	*r*(H^β^–C^β^)	*r*(C^α^–C^β^)
0	1.249	1.950	1.377	1.458	1.310	1.911	1.330	1.470
1	1.171	2.045	1.464	1.433	1.146	2.032	1.508	1.435
2	1.102	2.009	1.582	1.442	1.132	2.051	1.533	1.430
3	1.130	2.148	1.519	1.412	1.120	2.077	1.552	1.425
4	1.114	2.164	1.546	1.411	1.159	2.054	1.500	1.428

#### Activation strain analysis

The above analyses show that a higher distortion of the substrate is connected with a higher barrier (E2 higher than S_N_2) and also that a poorer donor–acceptor interaction capability of the nucleophile results in higher barriers (higher barriers upon adding solvent molecules). To gain more quantitative insight into this, we performed activation strain analyses that decompose the reaction energy barrier (Δ*E*^‡^) relative to separate reactants into the activation strain (Δ*E*_strain_) and the TS interaction (Δ*E*_int_), as shown below (for details, see the ESI†):^80,84,85^Δ*E*^‡^ = Δ*E*_strain_ + Δ*E*_int_

As shown in Fig. S9a and S9b (ESI†), the destabilizing strain energy of the E2 path is significantly larger than that of the S_N_2 path. The reason is the aforementioned larger characteristic distortion associated with the E2 path in which two bonds are breaking (C^α^–Cl and C^β^–H) in the substrate as compared to the lesser characteristic distortion associated with the S_N_2 path along which only one bond (C^α^–Cl) is breaking in the substrate.^86^ The higher activation strain is what makes the E2 barrier higher than the S_N_2 barrier, and this can only be inverted if the stabilizing nucleophile–substrate interaction is strong enough. As pointed out by Bickelhaupt *et al.*,^86^ the E2 pathway goes with a higher TS acidity, *i.e.*, the lower LUMO in the TS, than the S_N_2 pathway. However, neither HOO^−^ nor HO^−^ are strong enough bases to cause an inversion of barrier heights as determined by the unfavorably high activation strain for E2 reactions involving the CH_3_CH_2_Cl substrate. In view of the fact that introducing solvent molecules makes the nucleophile an even poorer electron donor, the E2 barrier becomes higher relative to the S_N_2 barrier as the degree of solvation increases.

### α-Effect

The HOO^−^ anion is an α-nucleophile, featuring a lone-pair bearing heteroatom adjacent to the nucleophilic center. Its parent normal nucleophile is HO^−^. The α-effect refers to the dramatically enhanced reactivity of α-nucleophiles compared to their parent normal nucleophiles by deviating downward from the Brønsted-type correlation (reaction barrier *versus* proton affinity) found for normal nucleophiles.^87^ To evaluate whether microhydrated HOO^−^ anions display an α-effect, two different Brønsted-type correlations between the barrier height and the basicity can be plotted by choosing different normal nucleophiles.

**Fig. 6:**
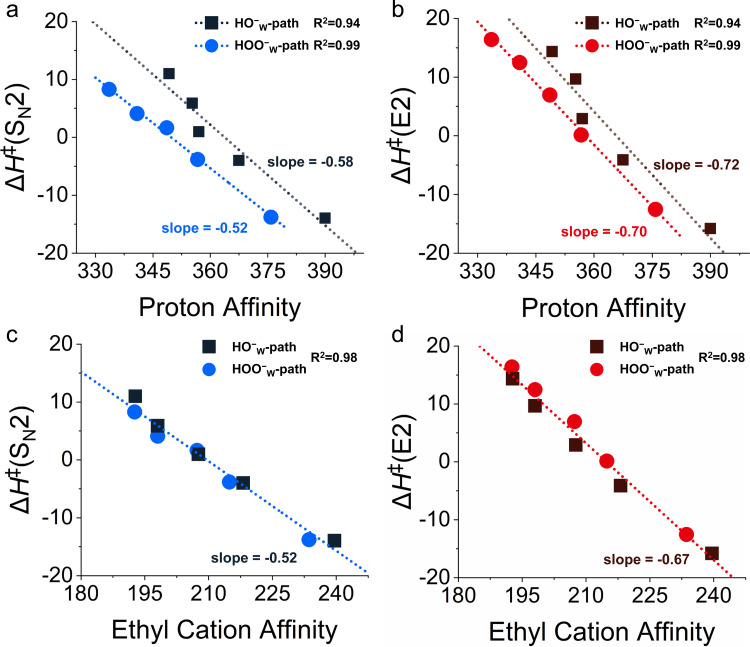
The correlation between HOO^−^(H_2_O)_*n*_ and HO^−^(H_2_O)_*n*_ + CH_3_CH_2_Cl reaction barrier heights Δ*H*^‡^ and the (a) and (b) proton affinity or (c) and (d) ethyl cation affinity of nucleophiles. The barrier heights Δ*H*^‡^ (in kcal mol^−1^) have been computed at CCSD(T)/aug-cc-pVTZ//MP2/6-311++G(d,p). The PA and ECA of the nucleophiles have been computed at G3(MP2).

In the Brønsted-type I correlation, one plots reaction barriers (Δ*H*^‡^) against the proton affinity (PA) of HOO^−^(H_2_O)_*n*_ and HO^−^(H_2_O)_*n*_ for *n* = 0 to 4. We found that there is a good correlation between Δ*H*^‡^ and the PA of the normal nucleophiles HO^−^(H_2_O)_*n*_ for both S_N_2 (Fig. 6a) and E2 (Fig. 6b) reactions. Thus, nucleophiles with larger PA values have lower barriers and, therefore, a higher reactivity. All the points of HOO^−^(H_2_O)_*n*_ deviate downward from this correlation line, with deviation values ΔΔ*H*^‡^ = Δ*H*^‡^(HO^−^ Brønsted path PA) − ΔΔ*H*^‡^(HOO^−^) of more than 5 kcal mol^−1^, revealing the existence of the α-effect for microhydrated HOO^−^ anions. The barriers of E2 reactions are more sensitive to the PA, as reflected by the larger slope of the correlation lines.

**Fig. 7:**
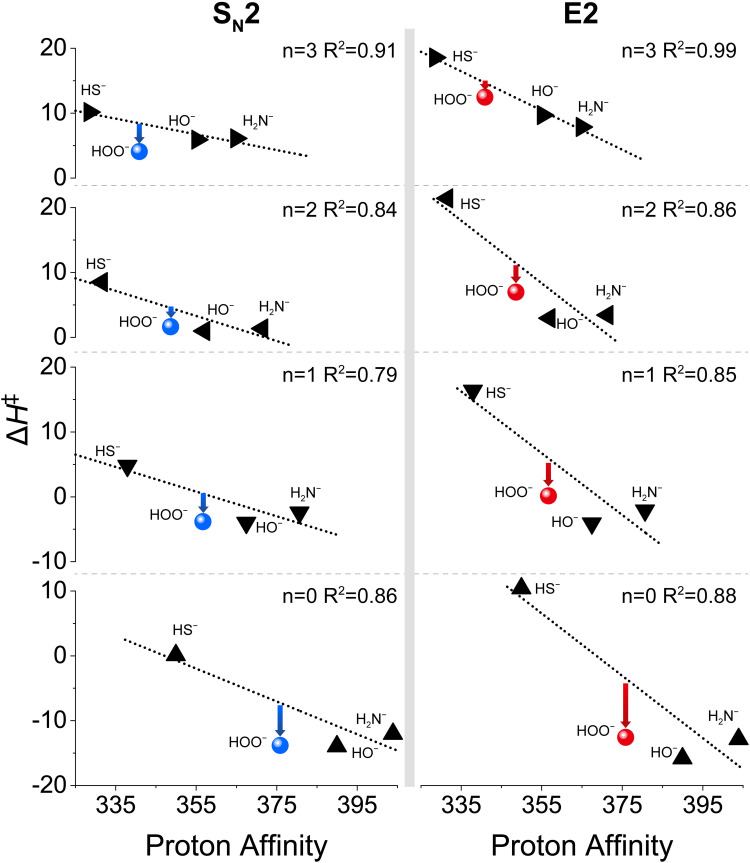
The correlation between HY^−^(H_2_O)_*n*_ (Y = O, S, HN) + CH_3_CH_2_Cl reaction barrier heights Δ*H*^‡^ and the proton affinity of the nucleophiles. The barrier heights Δ*H*^‡^ (in kcal mol^−1^) are obtained with the CCSD(T)/aug-cc-pVTZ method.

In the Brønsted-type II correlation, at each degree of hydration *n*, one plots the Δ*H*^‡^ of a set of other reference normal nucleophiles, including HO^−^, H_2_N^−^ and HS^−^, against the PA, and compares this with the barrier for the HOO^−^ anion and its PA. As shown in Fig. 7, when *n* = 0 to 3, the barriers of HOO^−^(H_2_O)_*n*_ show a downward shift from the Brønsted-type II Δ*H*^‡^*versus* PA correlation line of the normal nucleophiles for both S_N_2 and E2 reactions. This downward shift becomes less obvious for E2 when *n* = 1–3. This is consistent with the report of Hamlin *et al.*,^77^ where the unsolvated nucleophiles were considered reacting with ethyl halides. To our knowledge, no such correlation has been plotted for microsolvated nucleophiles reacting with ethyl halides, nevertheless, examples with methyl halides exist.^58,74,88–90^ Experimental studies by Bierbaum's group^58,74^ and computational investigations by Ren's group^88,90^ suggested that the α-effect exists for HOO^−^(H_2_O) and HOO^−^(CH_3_OH) reacting with methyl chloride. Herein, we expand the exploration of this phenomenon to ethyl halides covering both S_N_2 and E2 reactions. In brief, the Brønsted-type II analyses also suggest that HOO^−^(H_2_O)_*n*_ nucleophiles display the α-effect as compared with their normal nucleophile counterparts.

The mechanism behind the α-effect in S_N_2 reactions of unsolvated nucleophiles has been recently studied by Hamlin *et al.*^77^ who identified two criteria an α-nucleophile needs to fulfill in order to show the α-effect: (1) a small HOMO lobe and overall reduction of occupied amplitude on the nucleophilic center, in order to reduce the repulsive occupied–occupied orbital overlap between the nucleophile and the substrate and (2) a sufficiently high HOMO energy level, in order to still engage in a strong HOMO_nucleophile_–LUMO_substrate_ orbital interaction with the substrate. Herein, we examine whether the microsolvated HOO^−^(H_2_O)_*n*_ nucleophiles satisfy the two criteria. First, as shown in Fig. S10 (ESI†), the key occupied orbitals of nucleophiles, the HOMO lobes of HOO^−^(H_2_O)_*n*_, are smaller than those of HO^−^(H_2_O)_*n*_ and HO^−^(HOOH)(H_2_O)_*n*−1_ for nearly all degrees of microsolvation. This is further confirmed by the fact that the total negative charge on the nucleophilic center is significantly lower in the α-nucleophiles than in the corresponding normal nucleophile (see Fig. S1, ESI†). Second, if a DFT method is used, the HOMO levels of HOO^−^(H_2_O)_*n*_ are consistently at higher energy than those of HO^−^(H_2_O)_*n*_ and HO^−^(HOOH)(H_2_O)_*n*−1_. So, both criteria^77^ are indeed satisfied also in the case of microsolvated α-nucleophiles HOO^−^(H_2_O)_*n*_. Accordingly, the microsolvated HOO^−^(H_2_O)_*n*_ anion shall display an α-effect, consistent with the result given by Brønsted-type correlations.

Our above analyses show that the rate-accelerating α-effect in microsolvated E2 and microsolvated S_N_2 reactions goes hand-in-hand with a rise in orbital energy of the π-antibonding HOMO and the reduced amplitude of density on the nucleophilic center. The mechanism behind this is that the α-nucleophile has, due to its reduced density on the nucleophilic center, less steric (Pauli) repulsion with the substrate than the normal nucleophile and, therefore, a more stabilizing overall interaction;^77^ this difference in Pauli repulsion does not occur for the proton affinity because the proton has no occupied orbitals. However, if the ethyl cation affinity (ECA) instead of the PA is used as the Brønsted-correlation parameter, the α-effect is diminished; that is, the downward deviation of the barriers for the α-nucleophiles from the barrier *versus* the ECA Brønsted-type correlation is significantly reduced because the Pauli-reduction lowering in the case of α-nucleophiles relative to normal nucleophiles now happens not only in the interaction with the substrate in the TS of the reaction but also in the interaction with the carbon acid CH_3_CH_2_^+^ which defines the ECA. We had found this previously for S_N_2 reactions of unsolvated α-nucleophiles.^77^

Herein, we have been able to extend this finding to microsolvated nucleophiles and to E2 reactions. Thus, the barriers *versus* ECA correlations were constructed (Fig. S12d, ESI†), and the degree of downward deviation is indeed greatly reduced. In fact, as shown in Fig. 6c and d, the Δ*H*^‡^ values of both the HOO^−^_W_-path (α-nucleophile) and the HO^−^_W_-path (normal nucleophile) have a good linear relationship with the ECA. This phenomenon of reduced deviation is also observed when the HY^−^(H_2_O)_0–3_ is used as the reference, where Y = O, S, and HN (Fig. S15, ESI†), *i.e.* type II correlation. Altogether, our computed Brønsted-type correlations reveal that the microhydrated HOO^−^(H_2_O)_0–4_ nucleophiles exhibit the α-effect in both S_N_2 and E2 reactions.

## Conclusions

We have computed highly accurate potential energy profiles for various E2 and S_N_2 pathways involved in the HOO^−^(H_2_O)_0–4_ + CH_3_CH_2_Cl reaction system, involving both HOO^−^ and HO^−^ as attacking nucleophiles, based on a correlated CCSD(T)/aug-cc-pVTZ//MP2/6-311++G(d,p) approach. Our work provides both a benchmark description and a unified conceptual framework for a collection of interesting kinetic and structural phenomena that occur in our chemically rich series of model reactions in which microsolvated HOO^−^ has a dual appearance due to the facile solvent-induced formation of microsolvated HO^−^.

The S_N_2 path dominates the E2 path in our model systems. Adding water molecules further enhances the dominance of the S_N_2 reaction. This is so for both nucleophiles, HOO^−^(H_2_O)_0–4_ and HO^−^(HOOH)(H_2_O)_0–3_. Thus, the E2 barrier rises further above the S_N_2 barrier with each additional water molecule. This trend emerges from the combination of two factors: (i) the S_N_2 mechanism is associated with a smaller characteristic distortion and thus less activation strain Δ*E*_strain_ than the E2 mechanism; (ii) therefore, as the nucleophile–substrate interaction Δ*E*_int_ is weakened due to microsolvation, the barrier for the E2 path rises faster than that for S_N_2 and the latter pathway becomes more dominant.

In the S_N_2 substitution, the initial HOO^−^ nucleophile is clearly more reactive than the associated solvent-induced HO^−^ nucleophile. But, in the E2 elimination, the difference in reactivity is significantly smaller, with HOO^−^ still being somewhat more reactive. Thus, we found that the HOO^−^(H_2_O)_0–4_ nucleophiles display the α-effect in both the S_N_2 and, to a lesser extent, also in the E2 reaction. We show that the microsolvated α-nucleophiles satisfy the earlier proposed criteria for the occurrence of the α-effect, namely, a higher-energy HOMO and less occupied amplitude on the nucleophilic center, as compared to the corresponding normal nucleophile.

Our present work provides a unified description and rationalization of the reaction potential energy surface (PES) and kinetic and structural phenomena determined by this PES. A next leap forward that we envisage is the exploration of the complex dynamics taking place on this mechanistically rich multi-mechanistic PES of the HOO^−^(H_2_O)_0–4_ + CH_3_CH_2_Cl reactions.

## Computational methods

All calculations were performed using the Gaussian 16 program.^91^ To find an accurate method, the MP2,^92^ B97-1,^93^ B3LYP^94^ and CAM-B3LYP^95^ methods were tested on the reaction enthalpies of HOO^−^ + CH_3_CH_2_Cl to form CH_3_CH_2_OOH + Cl^−^ and CH_2_ = CH_2_ + Cl^−^ + HOOH. It turns out that the MP2/6-311++G(d,p) and MP2/aug-cc-pVTZ level of theories gave the best agreement with experimental values (Table S1, ESI†). We selected the MP2/6-311++G(d,p) method to perform the geometry optimization and frequency calculations throughout this work, for it is less time-consuming than the aug-cc-pVTZ basis set, and to be consistent with our previous work.^38^ The nature of stationary points was confirmed by the frequencies under harmonic oscillator approximation, where energy minimum structures have no imaginary frequency and transition state structures have one imaginary frequency. The intrinsic reaction coordinate (IRC) calculations were performed for all transition states to ensure accuracy. On top of the geometries optimized with MP2/6-311++G(d,p) level of theory, single point calculations were performed using coupled cluster theory CCSD(T)^96^ with the aug-cc-pVTZ basis set.^97,98^ If not specified, the energies reported in this work are at the CCSD(T)/aug-cc-pVTZ//MP2/6-311++G(d,p) level of theory.

## Data availability statement

The data that support the findings of this study are available in the ESI† of this article.

## Conflicts of interest

The authors declare no competing financial interests.

## Supplementary Material

CP-026-D4CP00671B-s001

CP-026-D4CP00671B-s002
