# Supplementary material for: Solvent-induced dual nucleophiles and the α-effect in the SN2 versus E2 competition
Source: Phys Chem Chem Phys. 2024 Mar 15;26(15):11320–30. doi: 10.1039/d4cp00671b (PMC11022550; doi:10.1039/d4cp00671b)
Supplement: CP-026-D4CP00671B-s002 [file CP-026-D4CP00671B-s002.pdf]

Electronic Supplementary Material (ESI) for Physical Chemistry Chemical Physics.
This journal is © the Owner Societies 2024

Supporting Information

Solvent-Induced Dual Nucleophiles and the α-Effect in the S_N2 *versus* E2 Competition

Xiangyu Wu[a], F. Matthias Bickelhaupt*[b,c,d], Jing Xie*[a]

[a] X. Wu, Prof. Dr. J. Xie
Key Laboratory of Cluster Science of Ministry of Education, Beijing Key Laboratory of Photoelectronic / Electrophotonic Conversion Materials, School of Chemistry and Chemical Engineering, Beijing Institute of Technology, Beijing, 100081, China.
E-mail: Jing Xie, jingxie@bit.edu.cn

[b] Prof. Dr. F. M. Bickelhaupt
Department of Chemistry and Pharmaceutical Sciences, AIMMS, Vrije Universiteit Amsterdam, De Boelelaan 1108, 1081 HZ Amsterdam (The Netherlands)
E-mail: f.m.bickelhaupt@vu.nl

[c] Prof. Dr. F. Matthias Bickelhaupt
Institute for Molecules and Materials (IMM), Radboud University Nijmegen, Heyendaalseweg 135, 6525 AJ Nijmegen (The Netherlands)

[d] Prof. Dr. F. Matthias Bickelhaupt
Department of Chemical Sciences, University of Johannesburg, Auckland Park, Johannesburg 2006 (South Africa)

Table of Contents

Table S1 Comparison of reaction enthalpies for HOO^{-} + CH_3CH_2Cl using different methods **S1**

Table S2 Difference in overall barriers between anti-E2 and inv-S_N2 pathways of $HOO^{-}(H_2O)_n$ + CH_3CH_2Cl reactions with the electronic energy (E), enthalpy (H, 298.15 K) and Gibbs free energy (G, 298.15 K) **S2**

Table S3 Difference in overall barriers between HOO^{-}_{W} and HO^{-}_{PT} pathways of $HOO^{-}(H_2O)_n$ + CH_3CH_2Cl reactions with the electronic energy (*E*), enthalpy (*H*, 298.15 K) and Gibbs free energy (*G*, 298.15 K) **S3**

Table S4. Orbital energy of the HOMO of $HOO^{-}(H_2O)_{n=0\text{-}4}$ and of $HO^{-}(H_2O)_{n=0\text{-}4}$. **S4**

Table S5. Proton affinities (PA) and ethyl cation affinities (ECA) of anions. **S5**

Table S6. Distortion index (%$D^{‡}$) of transition states. **S6**

Table S7. NPA atom charges *q* for transition states of $HOO^{-}(H_2O)_n$ + CH_3CH_2Cl reactions. **S7**

Figure S1. Structures and relative energies ΔE of $HOO^{-}(H_2O)_{0\text{-}4}$ cluster. **S8**

Figure S2. Structures and relative energies ΔE (in kcal/mol) of various transition states of the HOO^{-}_{W}-S_N2 path of $HOO^{-}(H_2O)_{0\text{-}4}$ + CH_3CH_2Cl. **S9**

Figure S3. Structures and relative energies ΔE (in kcal/mol) of various transition states of the HOO^{-}_{W}-E2 path of $HOO^{-}(H_2O)_{0\text{-}4}$ + CH_3CH_2Cl. **S10**

Figure S4. Structures and relative energies ΔE (in kcal/mol) of various transition states of the HO^{-}_{PT}-S_N2 path of $HO^{-}(H_2O)_{0\text{-}4}$ + CH_3CH_2Cl. **S11**

Figure S5. Structures and relative energies ΔE (in kcal/mol) of various transition states of the HO^{-}_{PT}-E2 path of $HO^{-}(H_2O)_{0\text{-}4}$ + CH_3CH_2Cl. **S12**

Figure S6. Structures and relative energies ΔE of reactant complexes and product complexes. **S13**

Figure S7. Correlation of S_N2 and E2 reaction barrier heights $\Delta E^{‡}$ with charge asymmetry Δq(Cl–O) = q(Cl) – q(O). **S14**

Figure S8. The correlation between a) $HOO^{-}(H_2O)_{n=0\text{-}4}$ + CH_3CH_2Cl, b) $HO^{-}(HOOH)(H_2O)_{n=0\text{-}3}$ + CH_3CH_2Cl, c) $HO^{-}(H_2O)_{n=0\text{-}4}$ + CH_3CH_2Cl reaction barrier height $\Delta E^{‡}$ and HOMO energy level of nucleophiles. **S15**

Figure S9. Activation strain analysis of transition states for $HOO^{-}(H_2O)_{n=0\text{-}4}$ + CH_3CH_2Cl reaction. **S16**

Figure S10. Occupied frontier orbitals computed at MP2/6-311++G(d,p). **S17**

Figure S11. Correlation of $HOO^{-}(H_2O)_{n=0\text{-}4}$ + CH_3CH_2Cl reaction barrier height $\Delta H^{\ddagger}$ with PA. **S18**

Table S8. Deviation $\Delta\Delta H^{\ddagger}$ of reaction barriers $\Delta H^{\ddagger}$(HO⁻ Brønsted path PA) derived from the proton-affinity-based HO^{-}_{PT} and the HO^{-}_{W} Brønsted-correlation path from the actual $\Delta H^{\ddagger}(HOO^{-})$ values of $HOO^{-}(H_2O)_{n=0\text{-}4}$ + CH_3CH_2Cl. **S19**

Figure S12. Correlation of $HO^{-}(H_2O)_{n=0\text{-}4}$ + CH_3CH_2Cl reaction barrier height $\Delta H^{\ddagger}$ with ECA. **S20**

Table S9. Deviation $\Delta\Delta H^{\ddagger}$ of reaction barriers $\Delta H^{\ddagger}$(HO⁻ Brønsted path ECA) derived from the ethyl-cation-affinity-based HO^{-}_{PT} and the HO^{-}_{W} Brønsted-correlation path from the actual $\Delta H^{\ddagger}(HOO^{-})$ values of $HOO^{-}(H_2O)_{n=0\text{-}4}$ + CH_3CH_2Cl. **S21**

Figure S13. Correlation of $HOO^{-}(H_2O)_{n=0\text{-}4}$ + CH_3CH_2Cl reaction barrier height $\Delta H^{\ddagger}$ with PA or ECA. **S22**

Figure S14. Correlation of $HY^{-}(H_2O)_{n=0\text{-}3}$ (Y = O, S, HN) + CH_3CH_2Cl reaction barrier height $\Delta H^{\ddagger}$ with PA. **S23**

Table S10. Deviation $\Delta\Delta H^{\ddagger}$ of reaction barriers $\Delta H^{\ddagger}$(HY⁻ Brønsted path PA) derived from the proton-affinity-based HY⁻Brønsted-correlation path of $HY^{-}(H_2O)_{n=0\text{-}3}$ (Y = O, S, HN) + CH_3CH_2Cl from the actual $\Delta H^{\ddagger}(HOO^{-})$ values of $HOO^{-}(H_2O)_{n=0\text{-}3}$ + CH_3CH_2Cl. **S24**

Figure S15. Correlation of $HY^{-}(H_2O)_{n=0\text{-}3}$ (Y = O, S, HN) + CH_3CH_2Cl reaction barrier height $\Delta H^{\ddagger}$ with ECA. **S25**

Table S11. Deviation $\Delta\Delta H^{\ddagger}$ of reaction barriers $\Delta H^{\ddagger}$(HY⁻ Brønsted path PA) derived from the ethyl-cation-affinity-based HY⁻Brønsted-correlation path of $HY^{-}(H_2O)_{n=0\text{-}3}$ (Y = O, S, HN) + CH_3CH_2Cl from the actual $\Delta H^{\ddagger}(HOO^{-})$ values of $HOO^{-}(H_2O)_{n=0\text{-}3}$ + CH_3CH_2Cl. **S26**

Table S1 Comparison of reaction enthalpies (298.15 K, kcal/mol) for HOO^- + CH_3CH_2Cl using different methods.[a]

$$HOO^- + CH_3CH_2Cl \rightarrow CH_3CH_2OOH + Cl^- \quad 0a$$
$$HOO^- + CH_3CH_2Cl \rightarrow CH_2{=}CH_2 + HOOH + Cl^- \quad 0c$$

Methods	ΔH_r(0a)	ΔH_r(0c)	MUE
Expt.[b]	-45.8	-24.5	0.0
CCSD(T)/aug-cc-pVTZ	-46.0	-24.0	0.3
MP2/6-311++G(d,p)	-45.0	-25.4	0.9
MP2/aug-cc-pVDZ	-48.5	-27.9	3.0
MP2/aug-cc-pVTZ	-45.6	-25.1	0.5
B97-1/aug-cc-pVDZ	-47.9	-27.2	2.4
B97-1/aug-cc-pVTZ	-46.4	-27.1	1.6
B3LYP/6-311++G(d,p)	-47.2	-30.2	3.5
CAM-B3LYP/6-31+G(d,p)	-49.0	-28.6	3.5
CAM-B3LYP/6-311++G(d,p)	-49.8	-27.5	3.6

[a] All computational values were obtained in the present study.

[b] Reference for experiment formation enthalpies (in kcal/mol) of anions and neutral molecules in gas phase at 298.15 K

	$\Delta_f H$	reference
HOO^-	-24.9	*J. Am. Chem. Soc.* **1981**, *103*, 6262
Cl^-	-56.1	*J. Phys. Chem. Ref. Data, Monograph 9*, **1998**
CH_3CH_2Cl	-26.9	CRC Handbook of Chemistry and Physics, 85th edition
CH_3CH_2OOH	-41.5	*J. Phys. Chem.* **1996**, *100*, 8240–8249
$CH_2{=}CH_2$	12.6	*J. Phys. Chem. Ref. Data, Monograph 9*, **1998**
HOOH	-32.7	*J. Phys. Chem. Ref. Data, Monograph 9*, **1998**

Table S2 Difference in overall barriers (in kcal/mol) between anti-E2 and inv-S_N2 pathways of $HOO^{-}(H_2O)_n$ + CH_3CH_2Cl reactions with the electronic energy (*E*), enthalpy (*H*, 298.15 K) and and Gibbs free energy (*G*, 298.15 K).[a]

	n	$\Delta\Delta^{\ddagger} = \Delta^{\ddagger}$(anti-E2) – $\Delta^{\ddagger}$(inv-S_N2)		
		$\Delta\Delta E^{\ddagger}$	$\Delta\Delta H^{\ddagger}$	$\Delta\Delta G^{\ddagger}$
**HOO^{-}_{W}**	0	4.3	1.2	0.4
	1	7.0	4.0	2.7
	2	7.4	5.3	6.0
	3	10.8	8.4	6.8
	4	9.9	8.1	7.0
**HO^{-}_{PT}**	0	1.3	-1.8	-2.6
	1	5.2	1.9	0.1
	2	6.2	3.2	1.1
	3	7.2	4.5	2.6
	4	8.3	5.2	4.5

[a] Computed at CCSD(T)/aug-cc-pVTZ//MP2/6-311++G(d,p).

Table S3 Difference in overall barriers (in kcal/mol) between HOO^{-}_{W} and HO^{-}_{PT} pathways of $HOO^{-}(H_2O)_n$ + CH_3CH_2Cl reactions with the electronic energy (*E*), enthalpy (*H*, 298.15 K), and Gibbs free energy (*G*, 298.15 K).[a]

	n	$\Delta\Delta^{\ddagger} = \Delta^{\ddagger}(HO^{-}_{PT}) - \Delta^{\ddagger}(HOO^{-}_{W})$		
		$\Delta\Delta E^{\ddagger}$	$\Delta\Delta H^{\ddagger}$	$\Delta\Delta G^{\ddagger}$
**inv-S_N2**	0	0.1	-0.2	-1.5
	1	3.2	3.5	4.2
	2	2.1	2.1	3.1
	3	4.9	5.2	4.6
	4	2.0	2.7	4.2
**anti-E2**	0	-3.0	-3.3	-4.6
	1	1.4	1.4	1.7
	2	0.9	0.0	-1.8
	3	1.2	1.3	0.4
	4	0.4	-0.2	1.7
**ret-S_N2**	0	4.4	4.2	1.9
**syn-E2**	0	-2.2	-2.6	-4.5

[a] Computed at CCSD(T)/aug-cc-pVTZ//MP2/6-311++G(d,p).

Table S4. Orbital energy (in eV) of the HOMO of $HOO^{-}(H_2O)_{n=0-4}$ and of $HO^{-}(H_2O)_{n=0-4}$[a]

n	$HOO^{-}(H_2O)_n$		$HO^{-}(HOOH)(H_2O)_n$		$HO^{-}(H_2O)_n$	
0	HOO^{-}	1.67 (-3.11)			HO^{-}	1.36 (-2.87)
1	$HOO^{-}(H_2O)$	[b]	$HO^{-}(HOOH)$	-0.99 (-5.40)	$HO^{-}(H_2O)$	-0.49 (-4.62)
2	$HOO^{-}(H_2O)_2$	-0.77 (-5.58)	$HO^{-}(HOOH)(H_2O)$	-1.66 (-6.49)	$HO^{-}(H_2O)_2$	-1.37 (-5.69)
3	$HOO^{-}(H_2O)_3$	-1.33 (-6.00)	$HO^{-}(HOOH)(H_2O)_2$	-2.35 (-6.89)	$HO^{-}(H_2O)_3$	-2.32 (-6.60)
4	$HOO^{-}(H_2O)_4$	-1.87 (-6.64)	$HO^{-}(HOOH)(H_2O)_3$	-2.78 (-7.28)	$HO^{-}(H_2O)_4$	-2.89 (-7.23)

[a] Values in normal text are computed at B97-1/6-311++G(d,p); values in parentheses are computed at HF/6-311++G(d,p)//MP2/6-311++G(d,p). [b] Spontaneous formation of $HO^{-}(HOOH)$.

Table S5. Proton affinities (PA) and ethyl cation affinities (ECA) of anions.[a]

n	$HOO^{-}(H_2O)_n$		$HO^{-}(HOOH)(H_2O)_n$		$HO^{-}(H_2O)_n$	
			PA ($Nu^{-} + H^{+} \rightarrow NuH$)			
0	HOO^{-}	375.9			HO^{-}	390.0
1	$HO^{-}(HOOH)$	356.7	$HO^{-}(HOOH)$	356.7	$HO^{-}(H_2O)$	367.4
2	$HOO^{-}(H_2O)_2$	348.6	$HO^{-}(HOOH)(H_2O)$	347.6	$HO^{-}(H_2O)_2$	357.0
3	$HOO^{-}(H_2O)_3$	340.9	$HO^{-}(HOOH)(H_2O)_2$	340.2	$HO^{-}(H_2O)_3$	355.3
4	$HOO^{-}(H_2O)_4$	333.6	$HO^{-}(HOOH)(H_2O)_3$	333.0	$HO^{-}(H_2O)_4$	349.2
			ECA ($Nu^{-} + CH_3CH_2^{+} \rightarrow CH_3CH_2Nu$)			
0	HOO^{-}	233.6			HO^{-}	239.6
1	$HO^{-}(HOOH)$	214.9[b]	$HO^{-}(HOOH)$	207.6	$HO^{-}(H_2O)$	218.1
2	$HOO^{-}(H_2O)_2$	207.3	$HO^{-}(HOOH)(H_2O)$	198.3	$HO^{-}(H_2O)_2$	207.6
3	$HOO^{-}(H_2O)_3$	198.0	$HO^{-}(HOOH)(H_2O)_2$	190.6	$HO^{-}(H_2O)_3$	198.0
4	$HOO^{-}(H_2O)_4$	192.6	$HO^{-}(HOOH)(H_2O)_3$	184.5	$HO^{-}(H_2O)_4$	192.8

[a] Computed at using G3(MP2).
[b] Formation of $CH_3CH_2OOH(H_2O)$

Table S6. Distortion index (%D‡) of transition states.[a,b]

	HOO⁻W-path			HO⁻PT-path		
		inv-S_N2-TS			anti-E2-TS	
n	%C^{α}Cl‡	%$H^{\beta}C^{\beta}$‡	%D‡	%C^{α}Cl‡	%$H^{\beta}C^{\beta}$‡	%D‡
0	18.2	-0.1	18.1	20.3	-0.3	20.0
1	21.8	-0.1	21.7	25.9	-0.3	25.6
2	23.3	-0.1	23.3	26.8	-0.3	26.5
3	25.8	0.0	25.8	28.0	-0.3	27.7
4	27.0	-0.1	27.0	26.9	-0.3	26.7
		anti-E2-TS			anti-E2-TS	
n	%C^{α}Cl‡	%$H^{\beta}C^{\beta}$‡	%D‡	%C^{α}Cl‡	%$H^{\beta}C^{\beta}$‡	%D‡
0	9.1	25.8	34.9	6.9	21.5	28.5
1	14.4	33.7	48.1	13.7	37.7	51.4
2	12.4	44.5	56.9	14.8	40.0	54.7
3	20.2	38.7	58.9	16.2	41.7	57.9
4	21.0	41.2	62.2	14.9	37.0	51.9

[a] Computed at MP2/6-311++(d,p).

[b] Definitions:

$$\%D^{\ddagger} = \%C^{\alpha}Cl^{\ddagger} + \%H^{\beta}C^{\beta\ddagger}$$

$$\%C^{\alpha}Cl^{\ddagger} = 100\times(r^{\ddagger}{}_{C^{\alpha}-Cl} - r^{Reactant}{}_{C^{\alpha}-Cl})/r^{Reactant}{}_{C^{\alpha}-Cl}$$

$$\%H^{\beta}C^{\beta\ddagger} = 100\times(r^{\ddagger}{}_{H^{\beta}-C^{\beta}} - r^{Reactant}{}_{H^{\beta}-C^{\beta}})/r^{Reactant}{}_{H^{\beta}-C^{\beta}}$$

Table S7. NPA atom charges *q* (in a.u.) for transition states of $HOO^{-}(H_2O)_n$ + CH_3CH_2Cl reactions.[a]

	**HOO⁻W-path**					**HO⁻PT-path**				
	**inv-S_N2-TS**					**inv-S_N2-TS**				
n	*q*(O)	*q*(C^{α})	*q*(Cl)	*q*(CH_3CH_2Cl)	Δ*q*(Cl–O)	*q*(O)	*q*(C^{α})	*q*(Cl)	*q*(CH_3CH_2Cl)	Δ*q*(Cl–O)
0	-0.656	0.012	-0.539	-0.142	0.117	-1.224	0.044	-0.580	-0.161	0.644
1	-0.636	0.056	-0.598	-0.159	0.038	-1.176	0.105	-0.663	-0.187	0.513
2	-0.618	0.065	-0.618	-0.172	0.000	-1.150	0.104	-0.672	-0.194	0.478
3	-0.651	0.091	-0.658	-0.178	-0.007	-1.138	0.111	-0.689	-0.202	0.449
4	-0.650	0.100	-0.675	-0.186	-0.025	-1.144	0.095	-0.667	-0.183	0.478
	**anti-E2-TS**					**anti-E2-TS**				
n	*q*(O)	*q*(H^{β})	*q*(Cl)	*q*(CH_3CH_2Cl)	Δ*q*(Cl–O)	*q*(O)	*q*(H^{β})	*q*(Cl)	*q*(CH_3CH_2Cl)	Δ*q*(Cl–O)
0	-0.617	0.417	-0.355	-0.221	0.262	-1.190	0.409	-0.313	-0.199	0.877
1	-0.608	0.451	-0.458	-0.254	0.150	-1.132	0.467	-0.447	-0.267	0.685
2	-0.613	0.472	-0.422	-0.292	0.192	-1.119	0.471	-0.465	-0.276	0.654
3	-0.659	0.480	-0.564	-0.268	0.096	-1.112	0.474	-0.492	-0.283	0.620
4	-0.643	0.481	-0.580	-0.284	0.063	-1.133	0.462	-0.466	-0.255	0.666

[a] Computed at MP2/6-311++G(d,p).

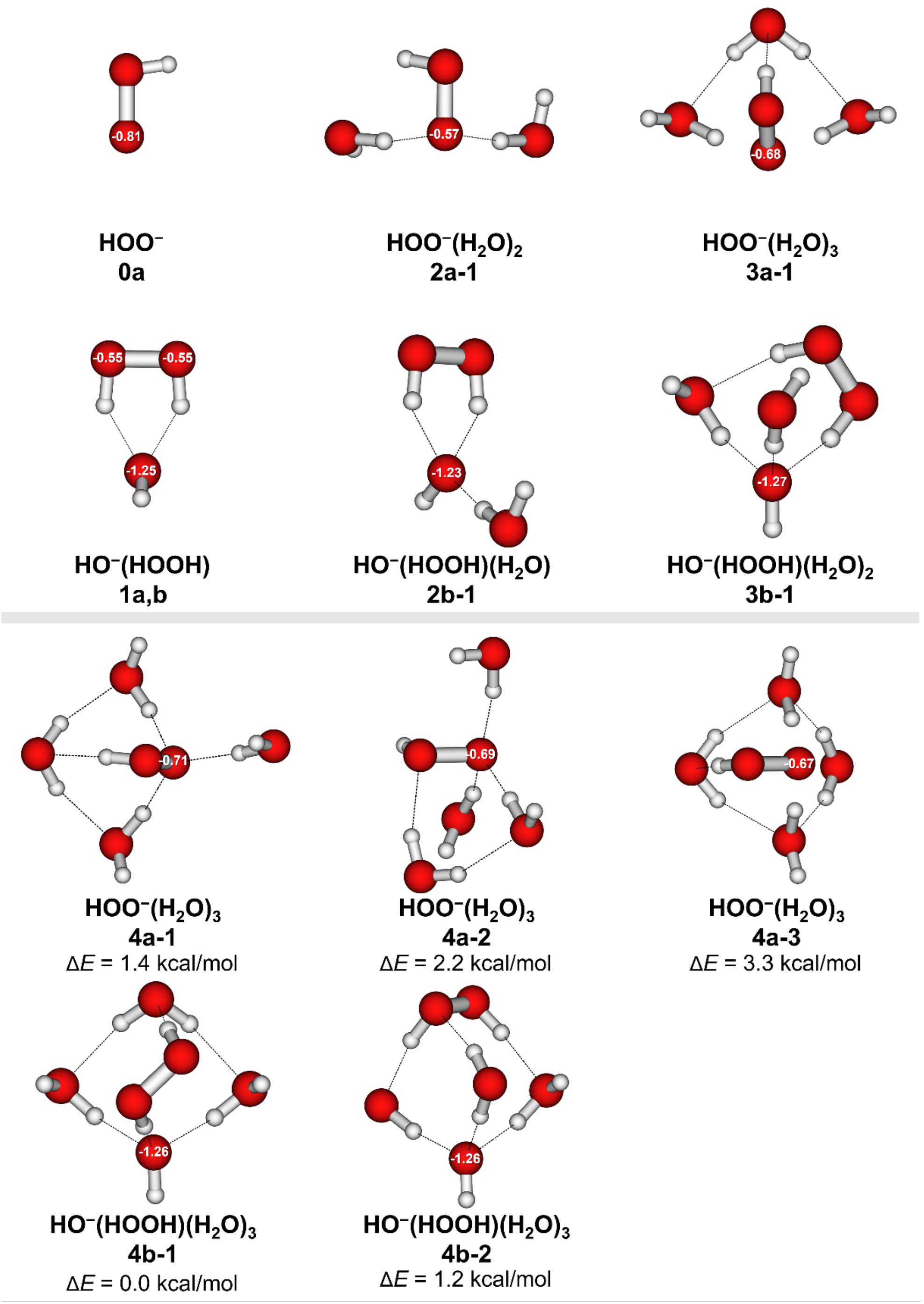

Figure S1. Structures and relative energies Δ*E* (in kcal/mol) of $HOO^{-}(H_2O)_{0\text{-}4}$ cluster. Energies Δ*E* for the **4a** and **4b** series relative to **4b-1**, computed at CCSD(T)/aug-cc-pVTZ//MP2/6-311++G(d,p) (without ZPE). The numbers represent the NPA charges (in a.u.) on the attacking O-atom.

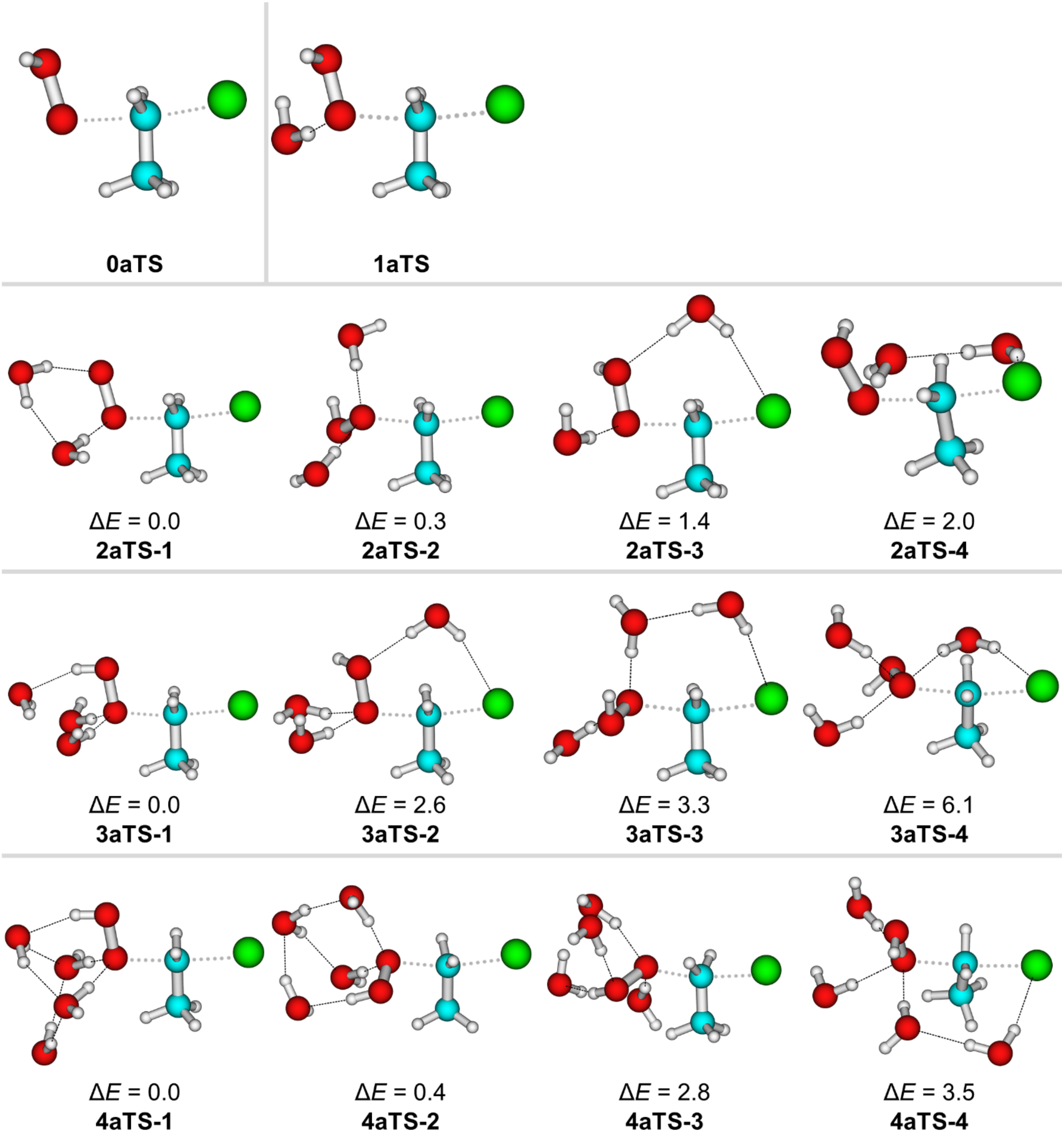

Figure S2. Structures and relative energies Δ*E* (in kcal/mol) of various transition states of the $HOO^{-}{}_{W}$-S_N2 path of $HOO^{-}(H_2O)_{0\text{-}4}$ + CH_3CH_2Cl. Energies Δ*E* are relative to **2aTS-1**, **3aTS-1**, or **4aTS-1**, respectively, and have been computed at MP2/6-311++G(d,p) (without ZPE).

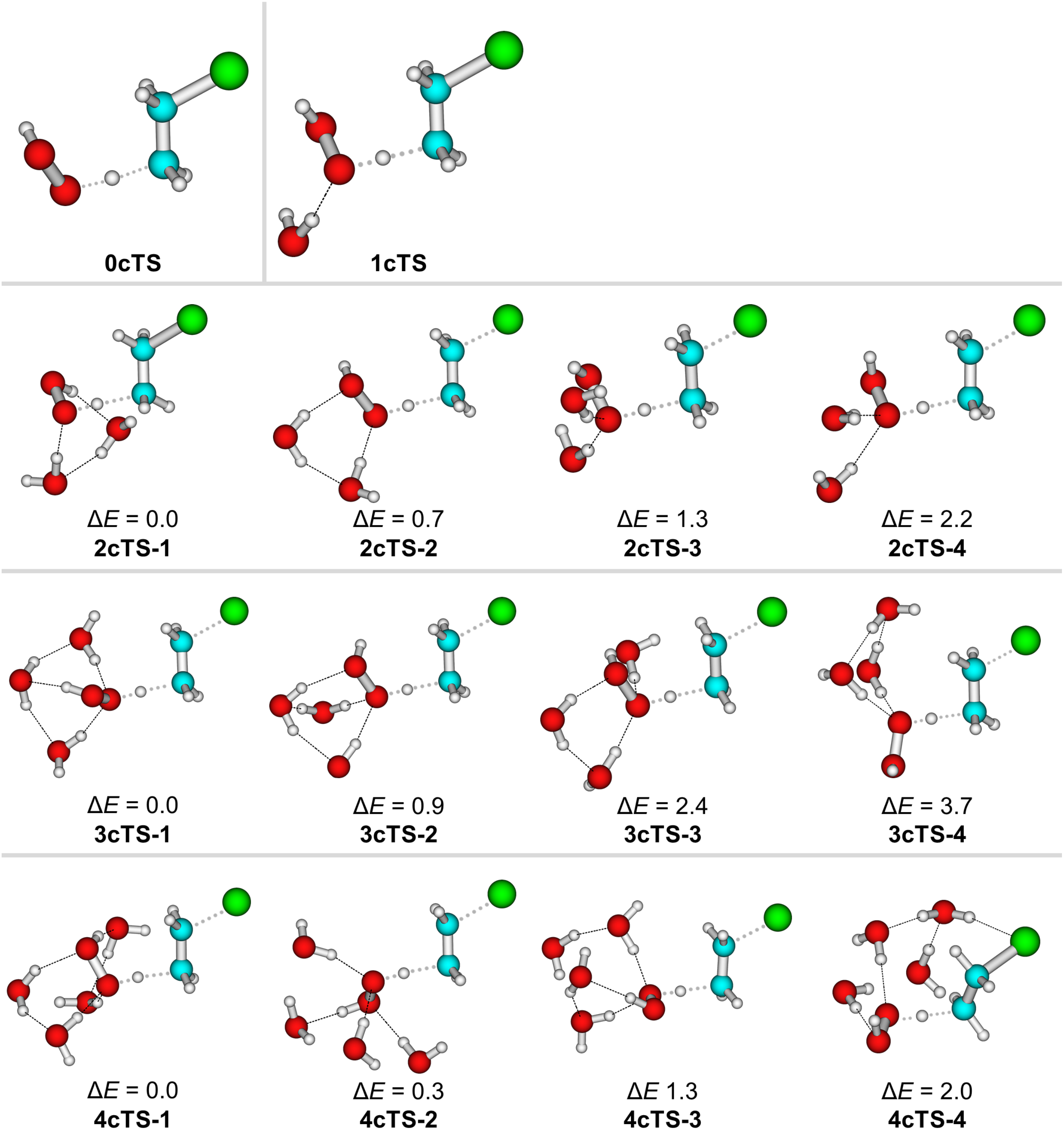

Figure S3. Structures and relative energies ΔE (in kcal/mol) of various transition states of the $HOO^{-}{}_{W}$-E2 path of $HOO^{-}(H_2O)_{0\text{-}4}$ + CH_3CH_2Cl. Energies ΔE are relative to **2cTS-1**, **3cTS-1**, or **4cTS-1**, respectively, and have been computed at MP2/6-311++G(d,p) (without ZPE).

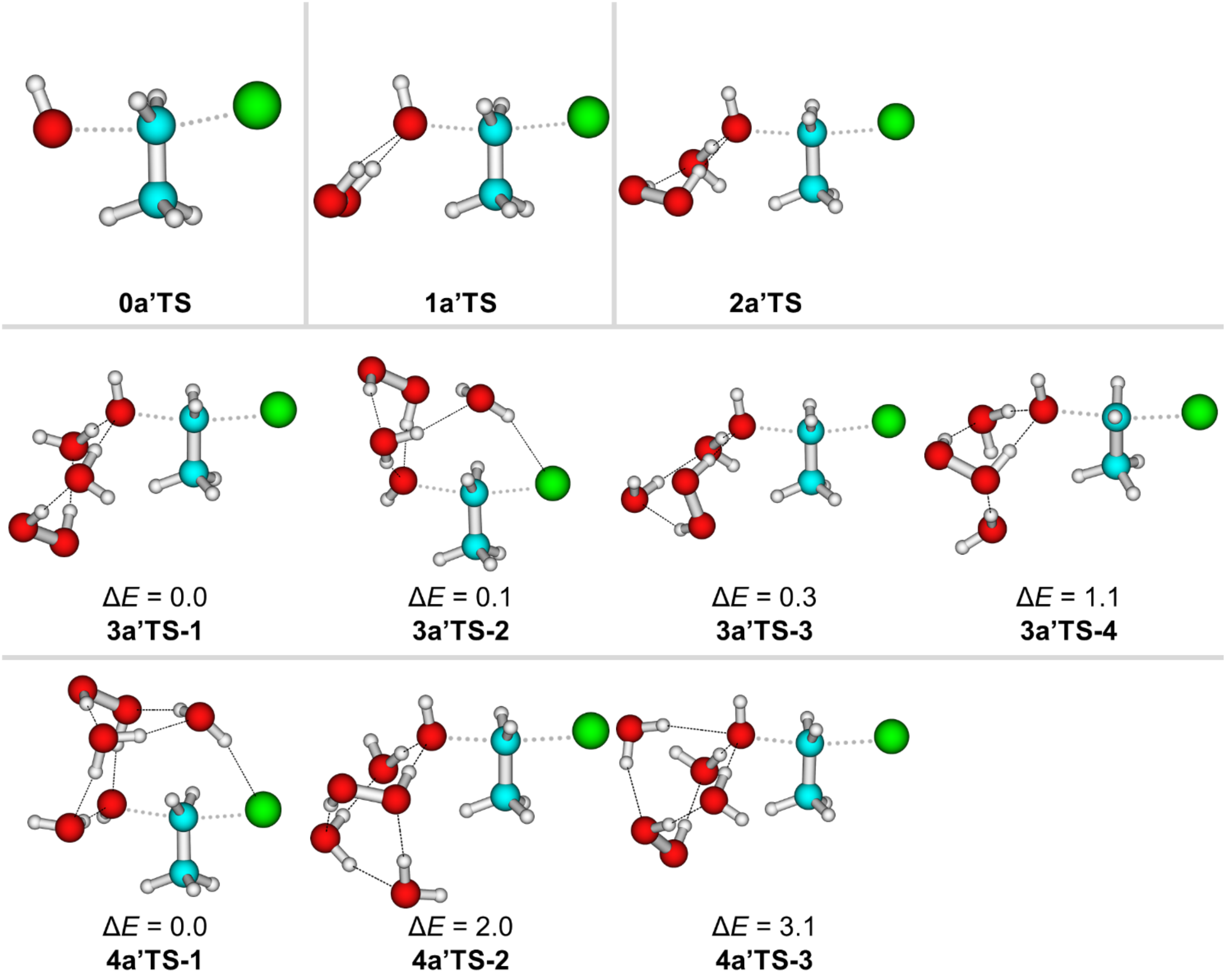

Figure S4. Structures and relative energies Δ*E* (in kcal/mol) of various transition states of the HO^{-}_{PT}-S_N2 path of $HO^{-}(H_2O)_{0\text{-}4}$ + CH_3CH_2Cl. Energies Δ*E* are relative to **3a'TS-1** or **4a'TS-1** and have been computed at MP2/6-311++G(d,p) (without ZPE).

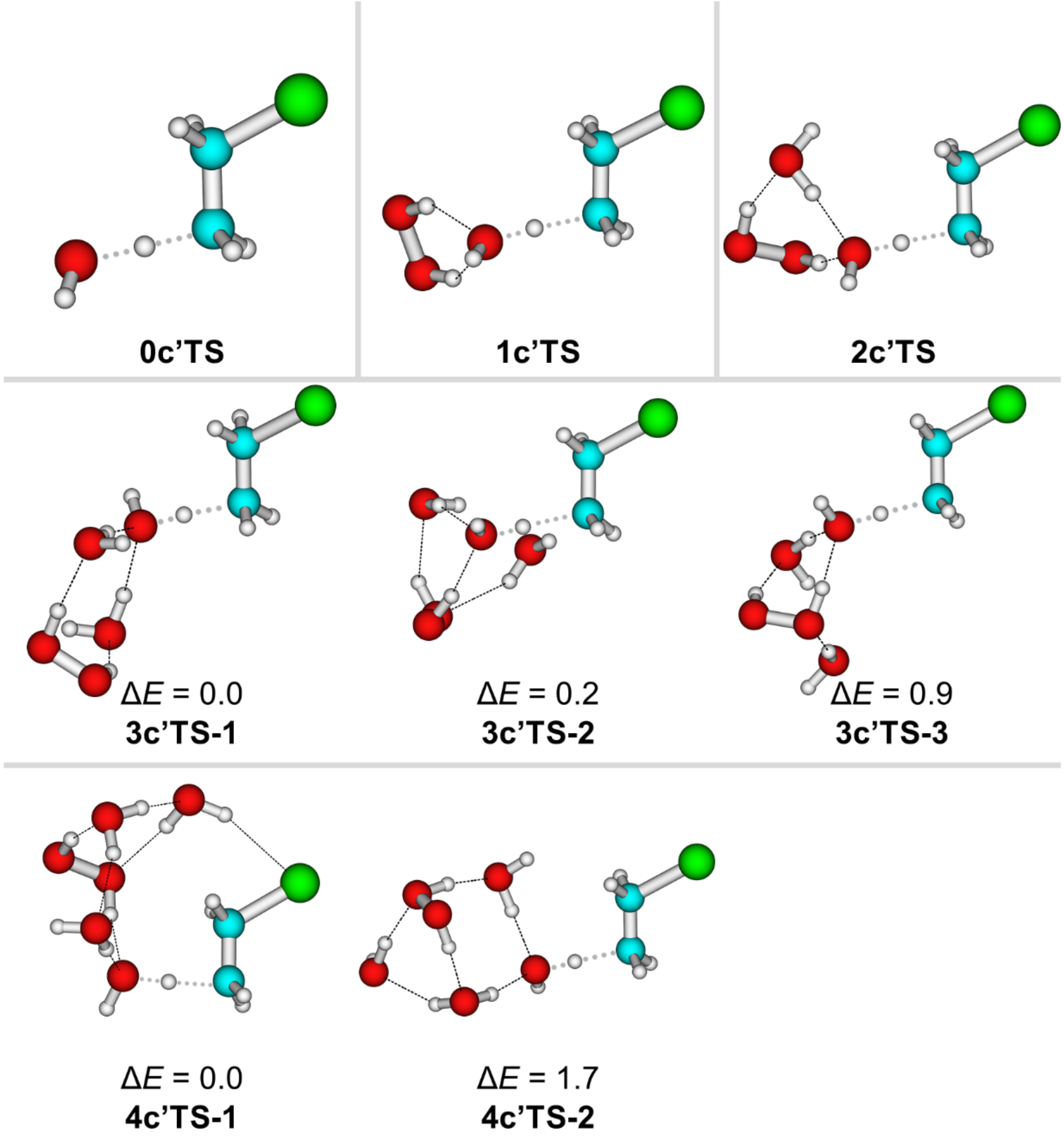

Figure S5. Structures and relative energies ΔE (in kcal/mol) of various transition states of the HO^{-}_{PT}-E2 path of $HO^{-}(H_2O)_{0\text{-}4}$ + CH_3CH_2Cl. Energies ΔE are relative to **3c'TS-1** or **4c'TS-1** and have been computed at MP2/6-311++G(d,p) (without ZPE).

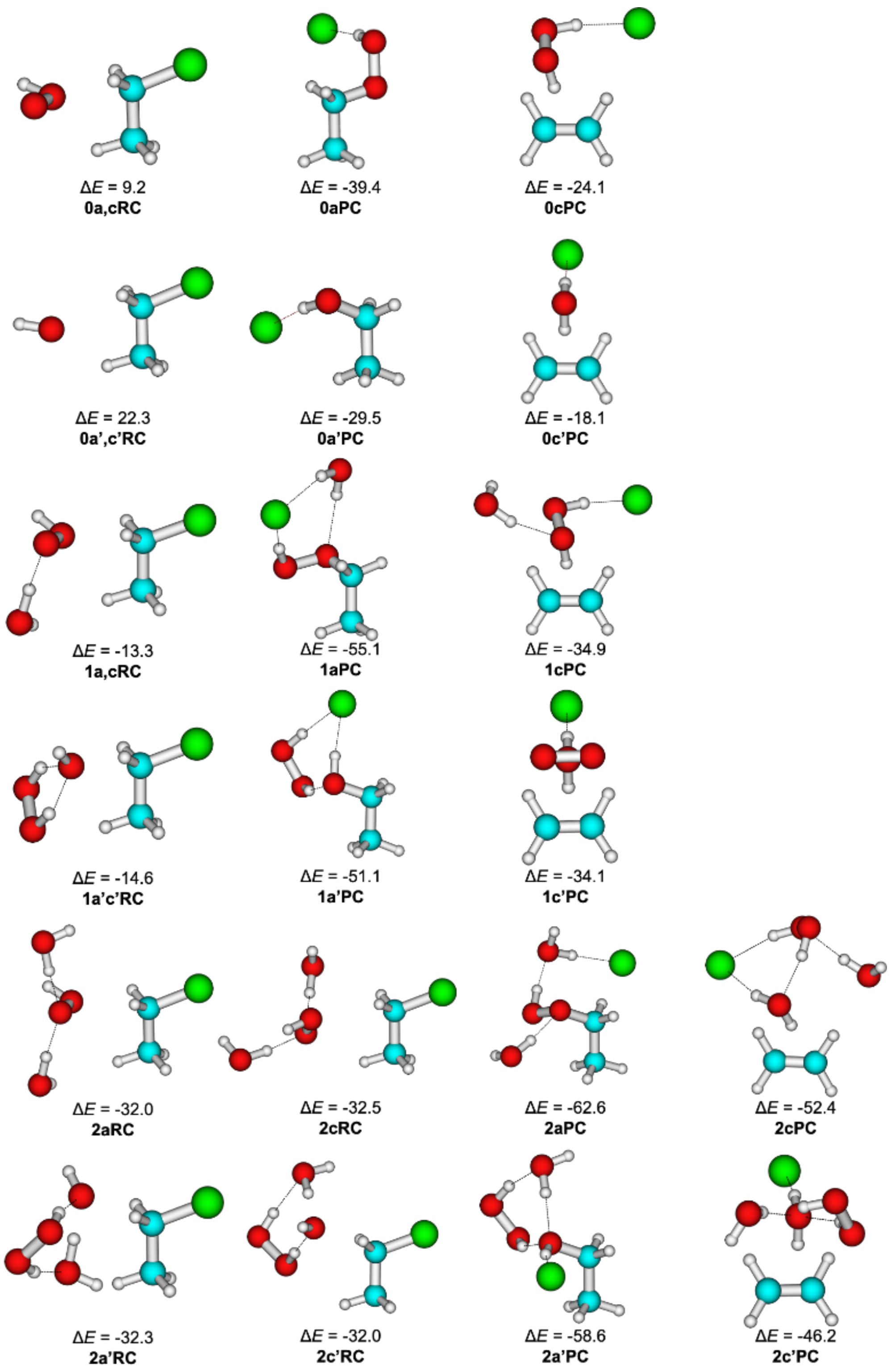

Figure S6. Structures and relative energies Δ*E* (in kcal/mol) of reactant complexes (RC) and product complexes (PC). Energies Δ*E* are relative to HO^{-}(HOOH) + CH_3CH_2Cl, computed at CCSD(T)/aug-cc-pVTZ//MP2/6-311++G(d,p) (without ZPE).

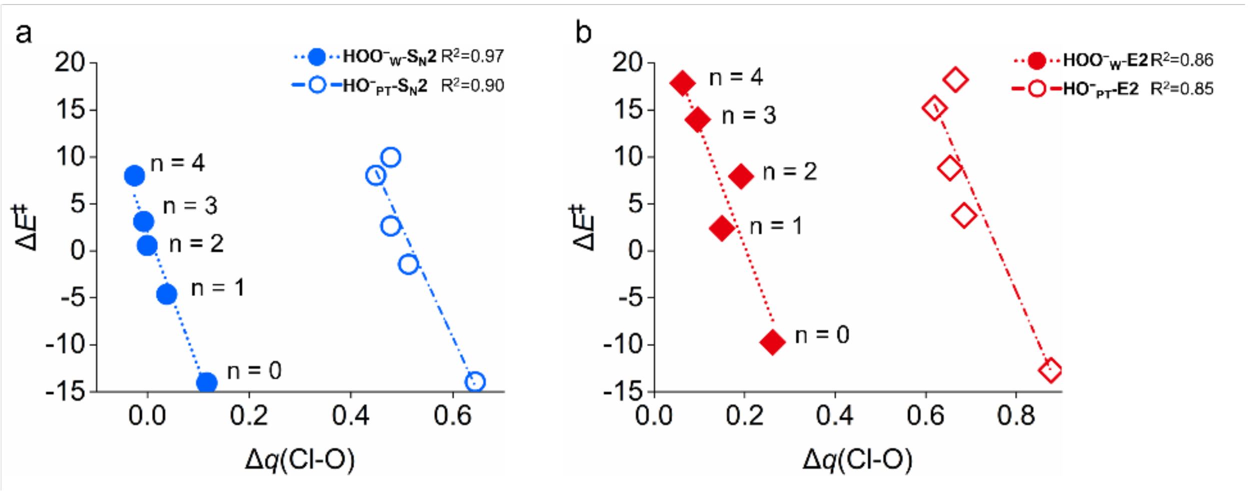

Figure S7. Correlation of S_N2 (a) and E2 (b) reaction barrier heights $\Delta E^{\ddagger}$ (in kcal/mol) with charge asymmetry Δq(Cl–O) = q(Cl) – q(O) (in a.u.) for the $HOO^{-}(H_2O)_{n=0\text{-}4}$ + CH_3CH_2Cl reaction, computed at CCSD(T)/aug-cc-pVTZ//MP2/6-311++G(d,p) (without ZPE). Note: for the HO^{-}_{PT}-pathway, n = 0 corresponds to the HO^{-} + CH_3CH_2Cl reaction.

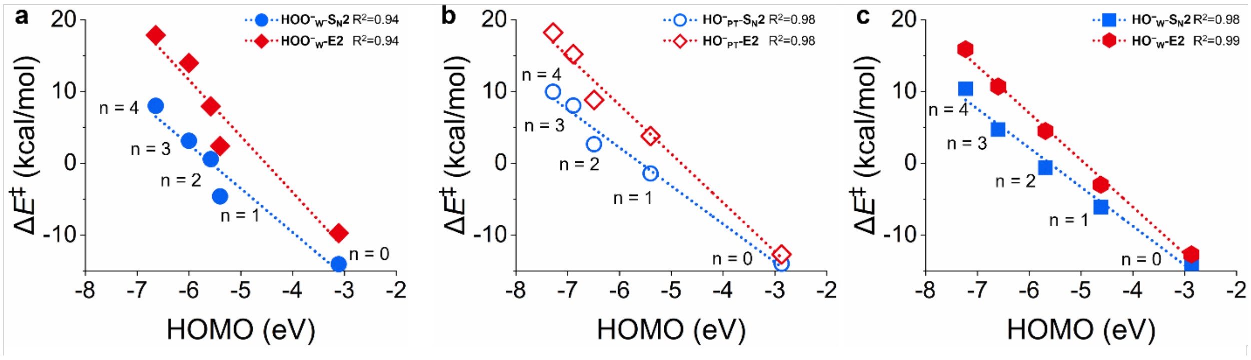

Figure S8. The correlation between (a) $HOO^{-}(H_2O)_{n=0\text{-}4}$ + CH_3CH_2Cl, (b) $HO^{-}(HOOH)(H_2O)_{n=0\text{-}3}$ + CH_3CH_2Cl, (c) $HO^{-}(H_2O)_{n=0\text{-}4}$ + CH_3CH_2Cl reaction barrier height $\Delta E^{\ddagger}$ (in kcal/mol) and HOMO energy level (in eV) of nucleophiles, computed at CCSD(T)/aug-cc-pVTZ//MP2/6-311++G(d,p) (without ZPE). Note: for the HO^{-}_{PT}-pathway, n = 0 corresponds to the HO^{-} + CH_3CH_2Cl reaction.

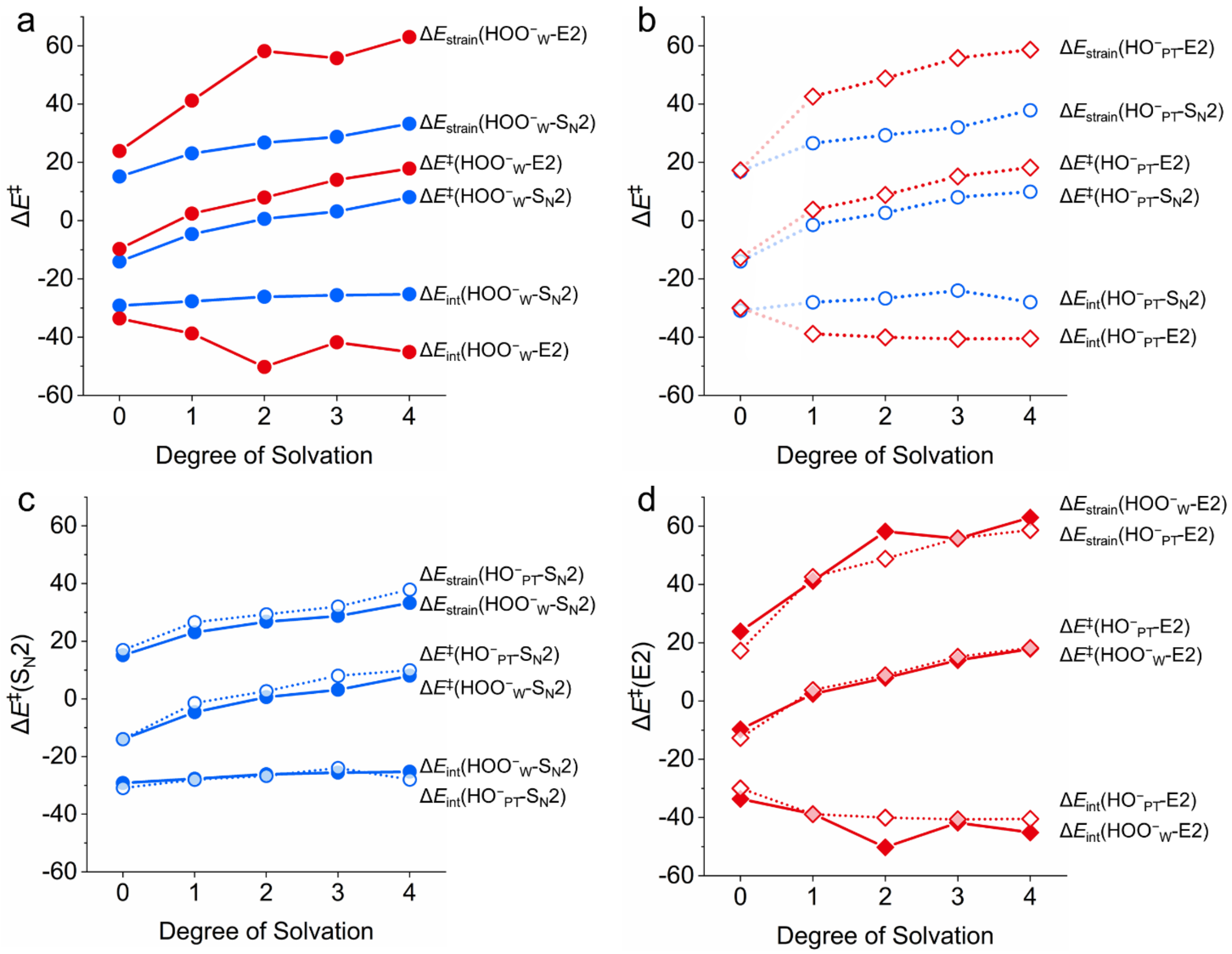

Figure S9. Activation strain analysis (in kcal/mol) of transition states for $HOO^{-}(H_2O)_{n=0\text{-}4}$ + CH_3CH_2Cl reaction, computed at CCSD(T)/aug-cc-pVTZ//MP2/6-311++G(d,p) (without ZPE).

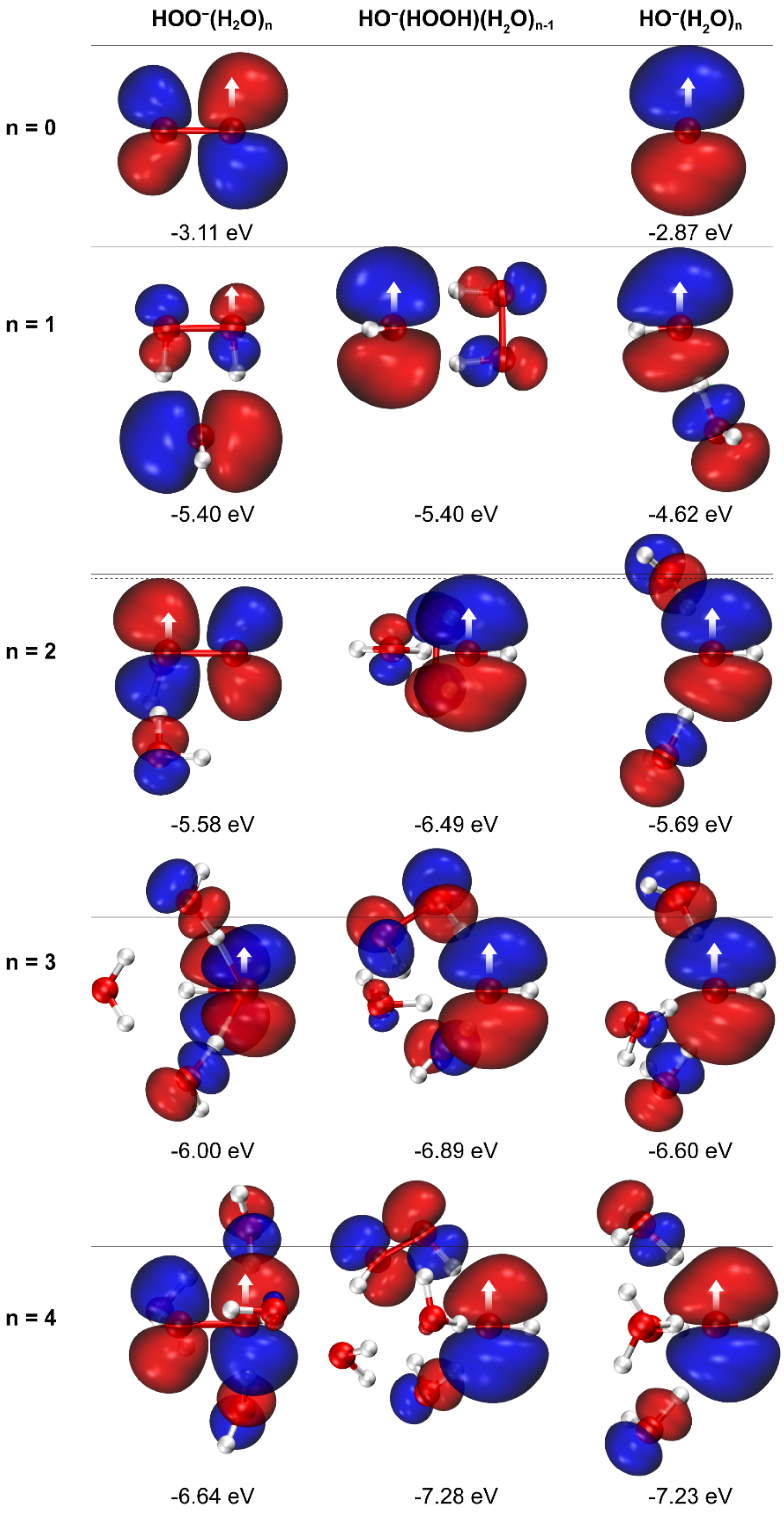

Figure S10. Occupied frontier orbitals, computed at MP2/6-311++G(d,p), using Gaussian 16[1] and Multiwfn[2] (isovalue = 0.03 bohr$^{-3/2}$).

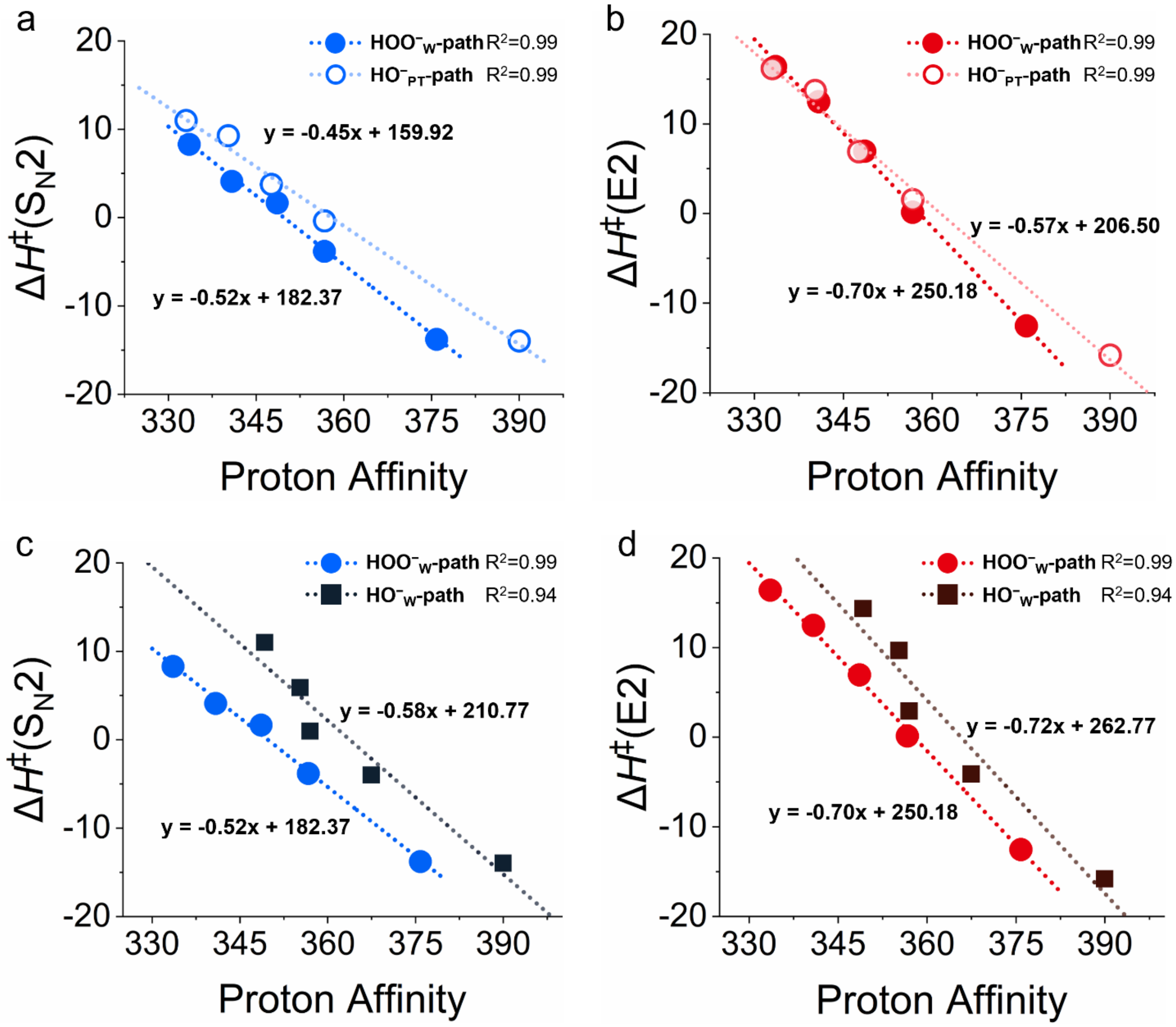

Figure S11. Correlation of $HOO^{-}(H_2O)_{n=0-4}$ + CH_3CH_2Cl reaction barrier height $\Delta H^{\ddagger}$ with PA (kcal/mol; S_N2 path = blue; E2 path = red), computed at CCSD(T)/aug-cc-pVTZ//MP2/6-311++G(d,p).The data of $HOO^{-}{}_{W}$-path can be found in Table S8.

Table S8. Deviation $\Delta\Delta H^{\ddagger}$ (in kcal/mol) of reaction barriers $\Delta H^{\ddagger}$(HO$^-$ Brønsted path PA) derived from the proton-affinity-based HO$^-_{PT}$ and the HO$^-_{W}$ Brønsted-correlation path from the actual $\Delta H^{\ddagger}$(HOO$^-$) values of HOO$^-$(H$_2$O)$_{n=0-4}$ + CH$_3$CH$_2$Cl.[a]

Path	n	$\Delta H^{\ddagger}$(HOO$^-$)	$\Delta H^{\ddagger}$(HO$^-$ Brønsted path PA)	$\Delta\Delta H^{\ddagger}$(deviation)
	0	-13.8	-8.0	5.8
	1	-3.8	0.5	4.3
HO$^-_{PT}$-S$_N$2	2	1.7	4.1	2.4
	3	4.1	7.6	3.5
	4	8.3	10.8	2.5
	0	-12.6	-8.3	4.3
	1	0.1	2.7	2.6
HO$^-_{PT}$-E2	2	6.9	7.3	0.4
	3	12.5	11.7	-0.8
	4	16.4	15.9	-0.5
	0	-13.8	-7.0	6.8
	1	-3.8	4.1	7.9
HO$^-_{W}$-S$_N$2	2	1.7	8.8	7.1
	3	4.1	13.2	9.1
	4	8.3	17.5	9.2
	0	-12.6	-7.3	5.3
	1	0.1	6.5	6.4
HO$^-_{W}$-E2	2	6.9	12.3	5.4
	3	12.5	17.8	5.3
	4	16.4	23.1	6.7

[a] Computed at CCSD(T)/aug-cc-pVTZ//MP2/6-311++(d,p). Graphical representation in Figure S11.

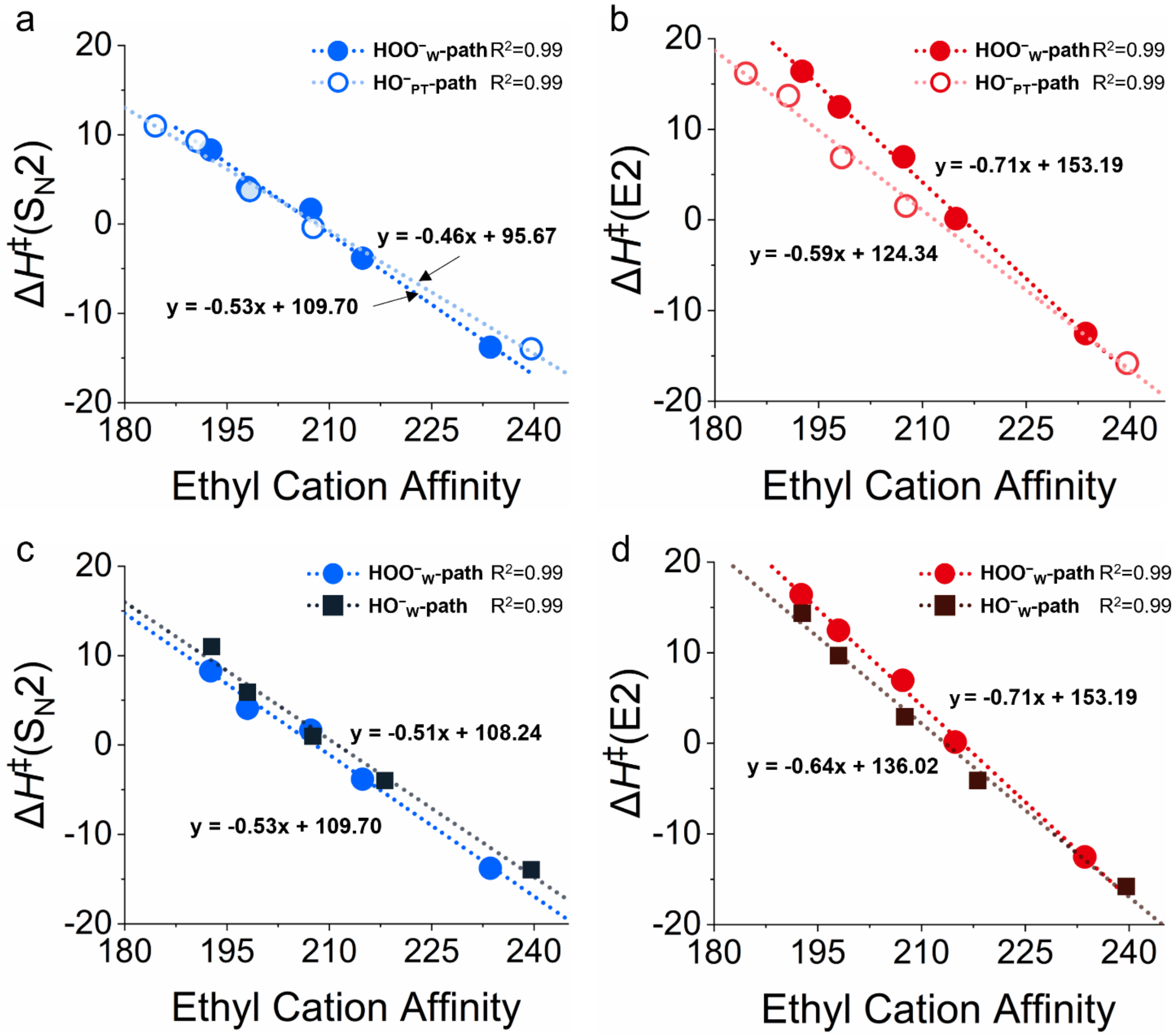

Figure S12. Correlation of $HO^{-}(H_2O)_{n=0-4}$ + CH_3CH_2Cl reaction barrier height $\Delta H^{\ddagger}$ with ECA (kcal/mol; S_N2 path =blue; E2 path = red), computed at CCSD(T)/aug-cc-pVTZ//MP2/6-311++G(d,p). The data of $HOO^{-}{}_{W}$-path can be found in Table S9.

Table S9. Deviation $\Delta\Delta H^{\ddagger}$ (in kcal/mol) of reaction barriers $\Delta H^{\ddagger}$(HO^- Brønsted path ECA) derived from the ethyl-cation-affinity-based HO^-_{PT} and the HO^-_{W} Brønsted-correlation path from the actual $\Delta H^{\ddagger}$(HOO^-) values of $HOO^-(H_2O)_{n=0-4}$ + CH_3CH_2Cl.[a]

Path	n	$\Delta H^{\ddagger}$(HOO^-)	$\Delta H^{\ddagger}$(HO^- Brønsted path ECA)	$\Delta\Delta H^{\ddagger}$(deviation)
HO^-_{PT}-S_N2	0	-13.8	-11.6	2.2
	1	-3.8	-3.0	0.8
	2	1.7	0.5	-1.2
	3	4.1	4.8	0.7
	4	8.3	7.2	-1.1
HO^-_{PT}-E2	0	-12.6	-12.8	-0.2
	1	0.1	-1.8	-1.9
	2	6.9	2.7	-4.2
	3	12.5	8.1	-4.4
	4	16.4	11.3	-5.1
HO^-_{W}-S_N2	0	-13.8	-11.5	2.3
	1	-3.8	-1.9	1.9
	2	1.7	2.0	0.3
	3	4.1	6.8	2.7
	4	8.3	9.5	1.2
HO^-_{W}-E2	0	-12.6	-12.9	-0.3
	1	0.1	-0.9	-1.0
	2	6.9	3.9	-3.0
	3	12.5	9.8	-2.7
	4	16.4	13.3	-3.1

[a] Computed at CCSD(T)/aug-cc-pVTZ//MP2/6-311++(d,p). Graphical representation in Figure S12.

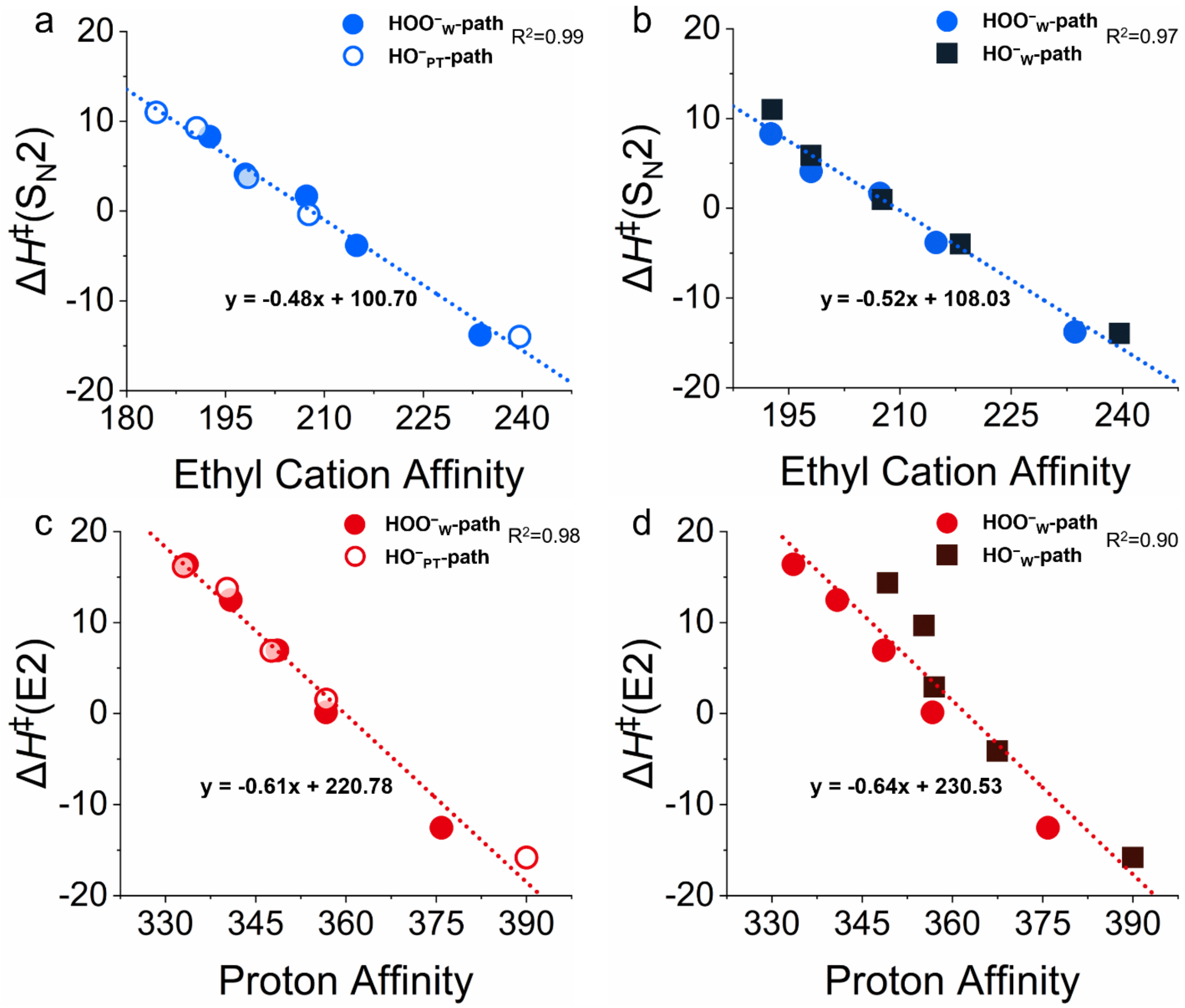

Figure S13. Correlation of $HOO^{-}(H_2O)_{n=0\text{-}4}$ + CH_3CH_2Cl reaction barrier height $\Delta H^{\ddagger}$ with PA or ECA (in kcal/mol; S_N2 path = blue; E2 path = red), computed at CCSD(T)/aug-cc-pVTZ//MP2/6-311++G(d,p).

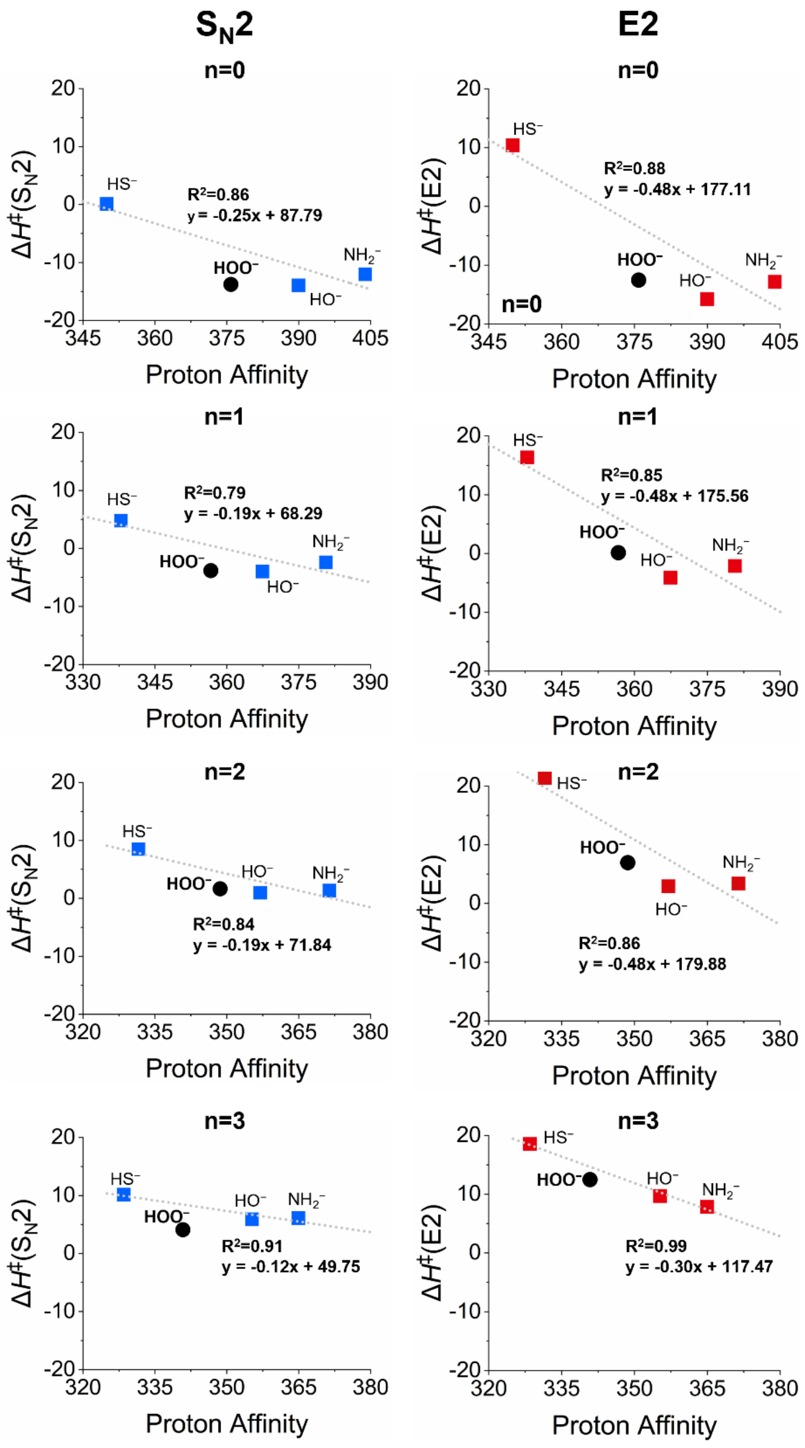

Figure S14. Correlation of $HY^{-}(H_2O)_n$ (Y = O, S, HN) + CH_3CH_2Cl reaction barrier height $\Delta H^{\ddagger}$ with PA (in kcal/mol; S_N2 path = blue; E2 path = red), computed at CCSD(T)/aug-cc-pVTZ//MP2/6-311++G(d,p). The data of the $HOO^{-}{}_{W}$-path can be found in Table S10.

Table S10. Deviation $\Delta\Delta H^{\ddagger}$ (in kcal/mol) of reaction barriers $\Delta H^{\ddagger}$(HY^{-} Brønsted path PA) derived from the proton-affinity-based HY^{-} Brønsted-correlation path of $HY^{-}(H_2O)_{n=0\text{-}3}$ (Y = O, S, HN) + CH_3CH_2Cl from the actual $\Delta H^{\ddagger}$(HOO^{-}) values of $HOO^{-}(H_2O)_{n=0\text{-}3}$ + CH_3CH_2Cl.[a]

n	Path	$\Delta H^{\ddagger}$(actual)	$\Delta H^{\ddagger}$(HO^{-} Brønsted path)	$\Delta\Delta H^{\ddagger}$(deviation)
0	S_N2-path	-13.8	-7.3	6.5
0	E2-path	-12.6	-3.5	9.1
1	S_N2-path	-3.8	0.5	4.3
1	E2-path	0.1	5.9	5.8
2	S_N2-path	1.7	4.5	2.9
2	E2-path	6.9	11.5	4.5
3	S_N2-path	4.1	8.4	4.3
3	E2-path	12.5	14.7	2.2

[a] Computed at CCSD(T)/aug-cc-pVTZ//MP2/6-311++(d,p). Graphical representation in Figure S14.

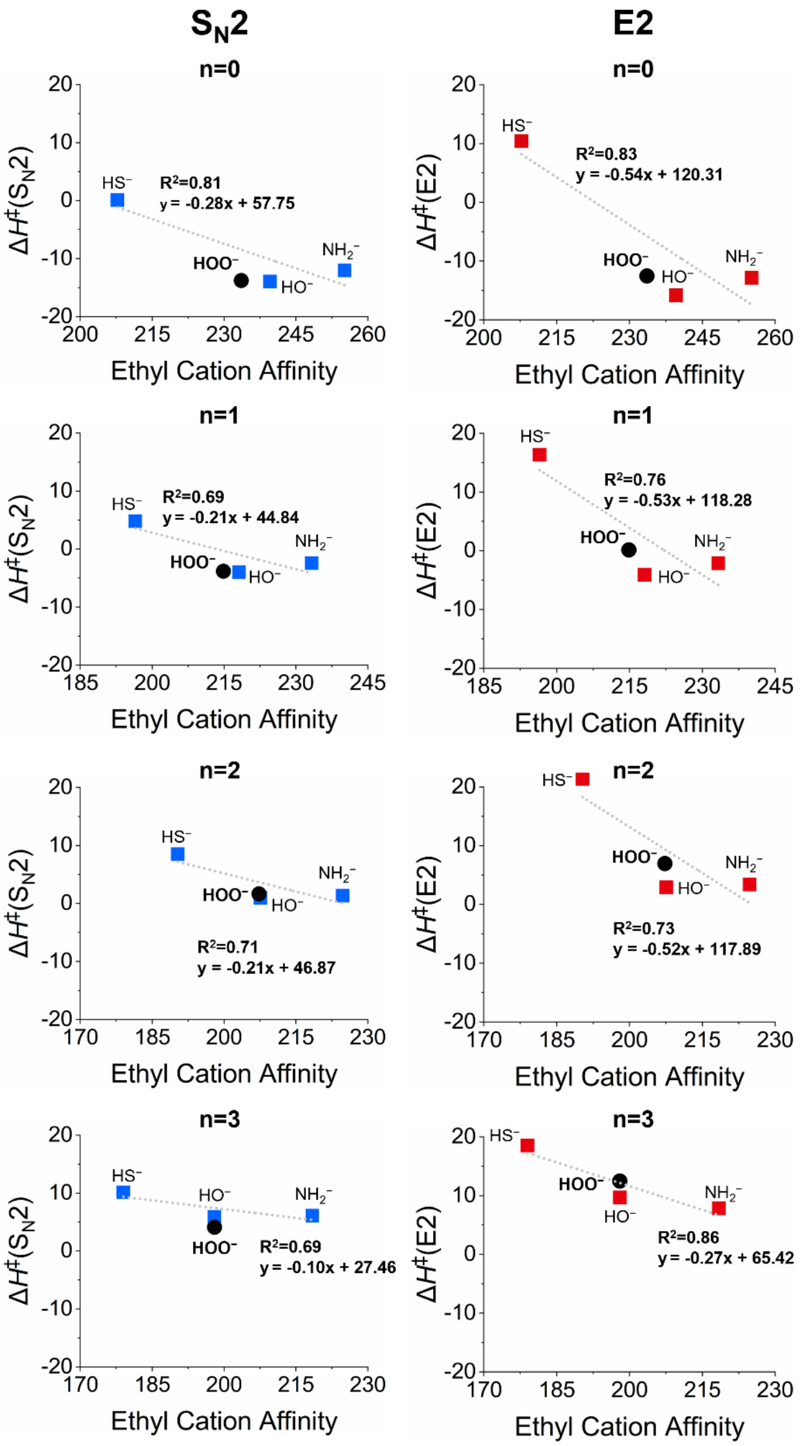

Figure S15. Correlation of $HY^{-}(H_2O)_n$ (Y = O, S, HN) + CH_3CH_2Cl reaction barrier height $\Delta H^{\ddagger}$ with ECA (in kcal/mol; S_N2 path =blue; E2 path = red), computed at CCSD(T)/aug-cc-pVTZ//MP2/6-311++G(d,p). The data of the HOO^{-}w-path can be found in Table S11.

Table S11. Deviation $\Delta\Delta H^{\ddagger}$ (in kcal/mol) of reaction barriers $\Delta H^{\ddagger}$(HY$^-$ Brønsted path ECA) derived from the ethyl-cation-affinity-based HY$^-$ Brønsted-correlation path of $HY^-(H_2O)_{n=0-3}$ (Y = O, S, HN) + CH_3CH_2Cl from the actual $\Delta H^{\ddagger}$(HOO$^-$) values of $HOO^-(H_2O)_{n=0-3}$ + CH_3CH_2Cl.[a]

n	Path	$\Delta H^{\ddagger}$(actual)	$\Delta H^{\ddagger}$(HO$^-$ Brønsted path)	$\Delta\Delta H^{\ddagger}$(deviation)
0	S_N2-path	-13.8	-8.5	5.3
0	E2-path	-12.6	-5.8	6.8
1	S_N2-path	-3.8	-0.3	3.5
1	E2-path	0.1	3.9	3.8
2	S_N2-path	1.7	3.7	2.0
2	E2-path	6.9	9.4	2.4
3	S_N2-path	4.1	7.4	3.3
3	E2-path	12.5	12.1	-0.3

[a] Computed at CCSD(T)/aug-cc-pVTZ//MP2/6-311++(d,p). Graphical representation in Figure S15.

References

[1] Gaussian 16 (Rev. A.03), M. J. Frisch, G. W. Trucks, H. B. Schlegel, G. E. Scuseria, M. A. Robb, J. R. Cheeseman, G. Scalmani, V. Barone, G. A. Petersson, H. Nakatsuji, X. Li, M. Caricato, A. V. Marenich, J. Bloino, B. G. Janesko, R. Gomperts, B. Mennucci, H. P. Hratchian, J. V. Ortiz, A. F. Izmaylov, J. L. Sonnenberg, Williams, F. Ding, F. Lipparini, F. Egidi, J. Goings, B. Peng, A. Petrone, T. Henderson, D. Ranasinghe, V. G. Zakrzewski, J. Gao, N. Rega, G. Zheng, W. Liang, M. Hada, M. Ehara, K. Toyota, R. Fukuda, J. Hasegawa, M. Ishida, T. Nakajima, Y. Honda, O. Kitao, H. Nakai, T. Vreven, K. Throssell, J. A. Montgomery Jr., J. E. Peralta, F. Ogliaro, M. J. Bearpark, J. J. Heyd, E. N. Brothers, K. N. Kudin, V. N. Staroverov, T. A. Keith, R. Kobayashi, J. Normand, K. Raghavachari, A. P. Rendell, J. C. Burant, S. S. Iyengar, J. Tomasi, M. Cossi, J. M. Millam, M. Klene, C. Adamo, R. Cammi, J. W. Ochterski, R. L. Martin, K. Morokuma, O. Farkas, J. B. Foresman, D. J. Fox, Gaussian, Inc., Wallingford, CT, **2016**.

[2] T. Lu, F. Chen, *J. Comput. Chem.* **2012**, *33*, 580.
